# Clickable Microgel Inks Enable Spatioselective, Multi‐Stimuli Programmable Assembly of Materials

**DOI:** 10.1002/advs.202520526

**Published:** 2026-01-30

**Authors:** Junho Moon, Frank Gardea, Madeline A. Morales, Svetlana Sukhishvili

**Affiliations:** ^1^ Department of Materials Science and Engineering Texas A&M University College Station Texas USA; ^2^ Army Research Directorate U.S. Army Combat Capabilities Development Command Army Research Laboratory South College Station Texas USA; ^3^ Army Research Directorate U.S. Army Combat Capabilities Development Command Army Research Laboratory Aberdeen Proving Ground Maryland USA

**Keywords:** 4D printing, Dynamic covalent bonds, Microgel assembly, Multi‐responsive hydrogels, Programmable materials, Spatioselective response

## Abstract

Life‐like materials that can dynamically morph their shape/texture, inspired by living organisms such as cephalopods are sought after for soft robotics and camouflage applications. Achieving such functions demands multimaterials with spatially programmed responses, yet their creation remains challenging. The existing approaches have been limited by the specific in situ polymerization conditions, fluidity of the synthetic components, and the complex, multi‐step processing requirements. Here, we propose a universal strategy that enables programming of material response within pre‐synthesized, ready‐to‐use, clickable microgels with different response behaviors. These microgels can be deposited within desired regions of the material structure via direct ink writing to enable pre‐programmed, localized responses to external stimuli. Spontaneous interparticle stabilization of the deposited microgels via a click reaction (Diels‐Alder bonding) yields shape‐stable, free‐form granular hydrogel multimaterials with reversible, repeatable, and spatially selective responses to stimuli (e.g., pH and temperature). The strategy establishes a 4D‐printing‐compatible, scalable modular platform for facile fabrication of soft materials with programmable shape adaptivity.

## Graphical abstract

Clickable microgel inks enable direct ink writing of hydrogel architectures with intrinsic spatioselective and programmable multi‐responsiveness. By combining pH‐responsive and temperature‐responsive microgel building blocks through Diels‐Alder interparticle crosslinking, the assemblies exhibit controllable swelling and shape changes. This modular platform illustrates a versatile strategy to design adaptive materials with tunable responses across multiple stimuli.

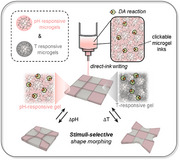

## 1 Introduction

Natural soft‐body organisms, such as cephalopods, octopuses, or plants can dynamically change their shape, texture, and function in response to a diverse range of environmental cues. Inspired by these features, synthetic life‐like hydrogel materials that can autonomously and reversibly respond to diverse environmental stimuli are developed for the next‐generation soft robotics, camouflage, and adaptive materials [1, 2, 3, 4]. However, achieving such behavior in synthetic materials has remained a challenge, as such life‐like materials require localized encoding of different/orthogonal functionalities, enabling different regions to undergo distinct, programmable transformations. Traditional responsive hydrogels are typically fabricated via in situ polymerization, resulting in mechanically robust and functionally uniform, bulk polymer networks [5]. This homogeneity and reliance on simplistic casting methods limit spatial control over local response and constrain the ability to achieve complex, multi‐step or directionally selective morphing [6, 7, 8]. To address these limitations, recent efforts have turned to 3D and 4D printing approaches to introduce structural and functional complexity into hydrogel systems.

4D printing refers to the fabrication of 3D objects that can change shape, function, or properties over time in response to external stimuli [9]. Traditional 4D materials are fabricated using extrusion‐based printing [10, 11] and vat photopolymerization [12, 13] (e.g., stereolithography and digital light processing), enabling unique design capabilities for hydrogel systems. These approaches have enabled hydrogel constructs to undergo programmable shape changes triggered by specific cues. In particular, extrusion‐based printing of hydrogel inks has enabled anisotropic shape morphing through patterned deposition of single‐material [14] or multi‐material inks [15, 16]. Despite these advances, most systems rely on precursor solutions containing monomers and rheology modifiers, which suffer from molecular diffusion during or after deposition [17]. This diffusion blurs functional interfaces, not only limiting the resolution of spatial patterning but effectively reducing the spatially diverse mechanical and morphing response. Furthermore, post‐curing steps such as UV exposure are typically required, limiting processing flexibility. As a result, only a few multi‐material 4D printed hydrogels have been reported, since these post‐processing steps restrict the ability to combine inks with very different properties (e.g., stimuli sensitivity) [18, 19, 20]. These challenges underscore the need for alternative strategies that allow spatially defined, multi‐functional responsiveness.

Granular hydrogels, constructed from discrete hydrogel microparticles or microgels, offer a compelling alternative for addressing the functional and processing challenges of traditional 4D printed hydrogels [21, 22]. Unlike monomeric precursor solutions that suffer from interfacial mixing, granular hydrogel systems are built from pre‐synthesized microgel units with defined size, chemistry, and responsiveness [23, 24]. This bottom‐up modularity allows physical patterning of microgels to construct 3D objects without relying on in situ polymerization. A few studies have demonstrated 4D printing of granular hydrogels to create architected structures with improved processability, often using mono‐responsive components [25, 26, 27, 28]. However, the ability to achieve locally programmable, orthogonal responsiveness within these assemblies remains limited. A key challenge lies in integrating robust interparticle cohesion that can endure multi‐stimuli conditions while still preserving the intrinsic responsive behavior of individual microgels. Recent strategies have introduced various interlinking chemistries including supramolecular interactions [29], dynamic covalent bonds [30, 31, 32, 33, 34, 35], and click chemistries [36, 37, 38] to create cohesive granular networks, but most of these linkages can dissociate upon environmental triggers such as pH or redox changes [32, 35]. Overcoming this trade‐off between structural integrity and functional specificity is essential to enable granular hydrogel systems capable of programmable, stimuli‐selective, life‐like shape transformations. A comparison of existing multi‐responsive hydrogel platforms is provided in Table S1, highlighting that no previously reported granular hydrogel system exhibits intrinsic multi‐stimuli actuation.

**FIGURE 1:**
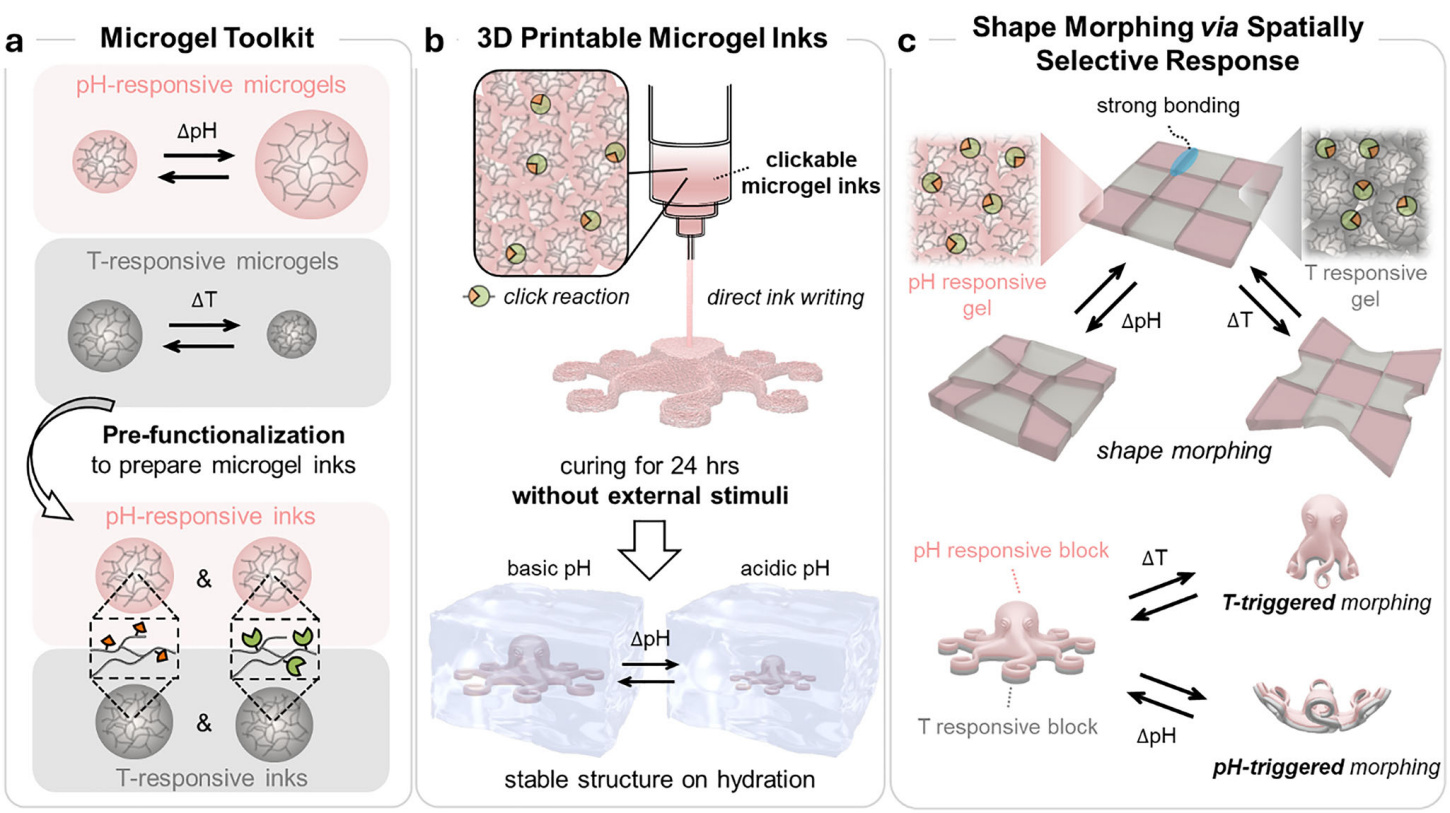
Schematic overview of the programmable multi‐responsive microgel assembly platform. (a) Design of a responsive microgel toolkit. Different microgel particles are programmed to respond to either pH or temperature. (b) Direct ink writing (DIW) of responsive structures using clickable microgel inks. Microgels with different responses spontaneously interconnect via click reaction after extrusion without the need for post‐curing treatments, forming strong multimaterial interfaces. (c) Spatial programming of the independent response of the materials’ domains to orthogonal environmental cues, enabling complex shape morphing in response to orthogonal stimuli.

In this work, we present a new modular concept for constructing shape morphing hydrogels with selectively programmable responses to multiple stimuli. This is achieved by assembling discrete microgels (components of a microgel toolkit), each programmed to respond to either temperature or pH cues (Figure 1a). These microgels spontaneously interconnect via click reaction, enabling structural cohesion and encoding of stimuli‐specific domains through direct ink writing (DIW, Figure 1b). Unlike previous approaches that rely on rheology modifiers or multi‐step post‐treatment [11, 39], our responsive, clickable ink allows precise spatial patterning of differential responses in a single processing step, offering a scalable and facile route for constructing programmable hydrogel architectures with stimuli‐selective shape morphing (Figure 1c).

## 2 Results and Discussion

### 2.1 Synthesis of a Stimuli‐Responsive Microgel Toolkit

**FIGURE 2:**
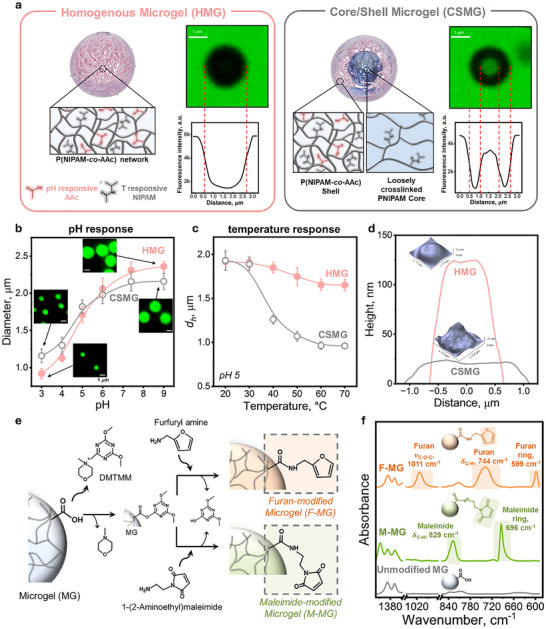
Design, characterization, and functionalization of pH‐ and temperature‐responsive microgel building blocks. (a) Schematic illustration and confocal microscopy images of homogeneous and core‐shell microgels (HMGs and CSMGs, respectively). Fluorescence intensity profiles indicate the spatial distribution of carboxylic acid groups, based on the exclusion of negatively charged fluorescein at pH 9. Scale bar is 1 µm. (b) pH‐responsive swelling of HMGs and CSMGs, quantified by fluorescence microscopy using fluorescein (acidic pH) and Rhodamine 6G (neutral and basic pH); the scale bar is 1 µm. See Figure S1 for the full set of images of microgels at different pHs. (c) Temperature‐responsive behavior characterized by hydrodynamic diameter, *d_h_*, measurements using DLS. (d) AFM images of dried HMG and CSMG particles deposited onto silicon wafers at pH 3, highlighting the differences in mechanical compliance and morphology. (e) Functionalization strategy introducing furan (F‐) and maleimide (M‐) groups onto microgels via DMTMM coupling chemistry. Functionalization was followed by five centrifugation‐resuspension in DI water cycles to remove unreacted reagents. (f) FTIR spectra of F‐, M‐, and unmodified HMG, confirming successful modification through characteristic vibrations of functional groups. The spectra were obtained after the samples were dried at the surface of a diamond ATR crystal by gently blowing N_2_ gas. FTIR spectra of F‐ and M‐HMGs modified at a coupling reagent‐to‐carboxylic acid molar ratio of 1:3.7 are shown here; data for other modification ratios and F‐/M‐ CSMGs are shown in Figure S5.

To enable programmable assembly and stimuli‐selective responsiveness, we designed two well‐defined microgels that respond to both pH and temperature, but with distinct response profiles arising from their internal structure. Specifically, we synthesized a homogeneous microgel (HMG) composed of a 1:1 molar ratio of N‐isopropylacrylamide (NIPAM) and acrylic acid (AAc) crosslinked with N,N′‐methylenebisacrylamide (Bis). The 1:1 NIPAM:AAc ratio was selected because it provides a balanced contribution of thermo‐responsive PNIPAM to assure successful synthesis of microgels using the nanoprecipitation techniques and ionizable AAc to support pH‐triggered swelling of HMG particles, while suppressing the lower critical solution temperature (LCST)‐type collapse of PNIPAM. As a result, HMG behaves primarily as a pH‐responsive microgel with minimal thermal contraction. We also synthesized a core‐shell microgel (CSMG) featuring a self‐crosslinked PNIPAM core and a shell comprising the same molar ratio of NIPAM:AAc crosslinked with Bis (Figure 2a). Here, “self‐crosslinking” refers to intramolecular and intermolecular chain‐transfer reactions of PNIPAM during precipitation polymerization, which generate short branching points and crosslinking even in the absence of an added crosslinker [40]. This produces lightly crosslinked, thermoresponsive PNIPAM network particles which serve as seeds for subsequent shell growth using NIPAM:AAc monomers. Both microgels were prepared by surfactant‐free nanoprecipitation using a semi‐batch monomer feeding method [41]. Confocal fluorescence microscopy with dye‐exclusion imaging at pH 9 revealed that carboxylic acid groups were uniformly distributed in HMG particles, whereas they were localized in the shell of CSMGs. At this pH, negatively charged fluorescein was repelled from the anionic microgel domains, resulting in dark circular microgels images  against a bright background. The average diameters of HMG and CSMG particles were ≈ 2.3 µm and ≈ 2.1 µm, respectively, with the CSMG shell estimated to be ≈ 0.55 µm thick. Both microgels displayed reversible pH‐responsive swelling from pH 3 to 9, but the extent of swelling was different depending on their structure (Figure 2b; Figure S1). HMGs exhibited a higher swelling ratio in response to pH change from pH 3 to 9 (≈ 2.5 ×), consistent with their higher content of ionizable carboxylic acid groups throughout the network, which promotes electrostatic repulsion upon deprotonation. In contrast, CSMGs showed a lower swelling ratio (≈ 1.9 ×) due to the presence of the microgel core that was not responsive to pH.

Temperature responsiveness of the dispersed microgels was evaluated by dynamic light scattering (DLS) (Figure 2c). HMG particles exhibited minimal change in hydrodynamic diameter, *d_h_*, with increasing temperature, due to suppression of LCST behavior typically observed in ionic NIPAM‐based polymers [42]. This suppression results from the balanced incorporation of hydrophilic AAc units, which offsets the phase separation caused by dehydration of NIPAM units. In contrast, CSMG particles showed an obvious decrease in *d_h_* above ≈ 32 °C, which is consistent with the LCST‐driven deswelling of its PNIPAM‐rich core. The continuous and coherent contraction during repeated thermal cycles suggests the presence of covalent links between the core and the shell domains that were created during the continuous feed polymerization (Figure S2). Together, these results establish that while both HMGs and CSMGs were dually responsive, their responsivity was selectively amplified by their structural designs: HMGs were predominantly pH‐responsive, while CSMGs primarily displayed temperature response.

To further probe the structural differences between HMGs and CSMGs beyond their swelling behavior in solution, the morphology of the dried microgels was characterized by atomic force microscopy (AFM) and scanning electron microscopy (SEM) (Figure 2d; Figure S3). Microgels were deposited onto a silicon wafer at pH 3 to ensure particle adhesion onto the silicon substrate. AFM height mapping revealed that dried HMG particles were thicker but did not show lateral spreading compared to CSMGs, suggesting a more robust, continuous internal network. In contrast, CSMG particles appeared more compliant and flattened, in agreement with the presence of a softer PNIPAM‐rich core that offers limited resistance to deformation upon drying. These observations provided further evidence of the structural differences between HMGs and CSMGs and are consistent with their respective design.

To enable programmable assembly, we next introduced reactive groups suitable for Diels‐Alder (DA) click chemistry to convert the microgels into functionalized components of the microgel toolkit. The furan‐maleimide DA reaction is a well‐established, catalyst‐free click reaction that proceeds rapidly under mild conditions [43], making it an ideal candidate for using the dynamic covalent chemistry in aqueous environment. Leveraging the carboxylic acid groups available in HMG and CSMG particles (0.11 and 0.08 mmol per mL of ≈ 1.7 wt.% microgel dispersions, respectively, as determined by the acid‐base titration; Figure S4), we employed 4‐(4,6‐dimethoxy‐1,3,5‐triazin‐2‐yl)‐4‐methylmorpholinium chloride (DMTMM) as a coupling agent to attach furan or maleimide moieties through the amide bond formation [44] (Figure 2e). This modification yielded a furan‐ and maleimide‐functionalized clickable microgel toolkit (F‐MGs and M‐MGs, respectively), capable of interparticle crosslinking upon mixing. Importantly, individual microgels were colloidally stable, and even mixing F‐MGs and M‐MGs at low‐salt aqueous dispersion did not trigger spontaneous crosslinking; the dispersions remained colloidally stable for over a month without aggregation. DA bond formation occurred only after microgels were brought into close contact during the processing steps described below in Section 2.2.  Thus, these functionalized microgels are chemically stable and “ready‐to‐use”, while interparticle DA bonding is triggered during processing and assembly. Successful incorporation of the reactive groups was confirmed by Fourier Transform Infrared (FTIR) spectroscopy (Figure 2f; Figure S5). F‐MGs showed characteristic peaks corresponding to the –C─O─C–stretching vibrations at 1011 cm^−1^, –C─H bending vibrations at 744 cm^−1^, and the furan ring bending vibrations at 599 cm^−1^ [45], while M‐MGs exhibited distinct absorption bands at 829 cm^−1^ (–C─H bending) and 696 cm^−1^ (maleimide ring bending vibrations) [46], indicative of maleimide incorporation. To examine the effect of reagent concentrations, FTIR spectra were collected at varying reagent‐to‐carboxylic acid molar ratios, showing progressive increases in the intensity of these characteristic peaks (Figure S5). Quantification was carried out by normalizing the peak areas (744 cm^−1^ –C─H bending for F‐MG and 696 cm^−1^ maleimide ring for M‐MG) to their respective saturated values (A/A_sat_). The resulting curves (Figure S6) revealed a clear dependence of the degree of modification on reagent loading, approaching a plateau at higher DMTMM/–COOH ratios. To simultaneously ensure sufficient microgel functionalization for following materials assembly with retention of free carboxyl groups necessary for microgel responsiveness, we adopted fixed coupling reagent‐to‐carboxyl molar ratios of 1:3.7 for HMGs and 1:2.7 for CSMGs, respectively. Under these conditions (corresponding to 0.27 and 0.36 reagent‐to‐COOH ratios for HMGs and CSMGs, respectively), the FTIR integration indicated ≈ 25 and ≈ 31% conversion of carboxyl groups for HMG and CSMG particles, respectively, suggesting effective functionalization of the microgels with furan or maleimide DA‐reactive groups.

To confirm the occurrence of interparticle crosslinking via DA chemistry, we mixed equal amounts of F‐MGs and M‐MGs and dried the mixed dispersion using vacuum‐assisted filtration. Rehydration of the film with water yielded stable, freestanding hydrogels. In contrast, a similar procedure applied to unmodified microgels resulted in a film which readily redispersed into individual particles (Figure S7). This indicates the formation of a covalent network between F‐MG and M‐MG particles, rendering the assembly insoluble. Further spectroscopic evidence of DA bond formation was obtained via FTIR analysis using dried films of HMG‐based modified microgels. The 696 cm^−1^ peak, associated with the maleimide ring bending vibrations in M‐MGs, disappeared upon crosslinking, while the furan C─H bending peak at 744 cm^−1^ shifted to 736 cm^−1^, indicating the formation of the DA adduct [47] (Figure S8). Additional confirmation of the DA bond formation was obtained by differential scanning calorimetry (DSC), which revealed a broad endothermic peak with an onset near 80 °C and a peak around 120 °C [48] in the DA‐crosslinked film (Figure S9). The latter thermal feature occurs due to the retro‐DA (rDA) dissociation process, further supporting the formation of the covalent linkages within the microgel assembly.

### 2.2 Rheology Control of Clickable Microgel Inks

**FIGURE 3:**
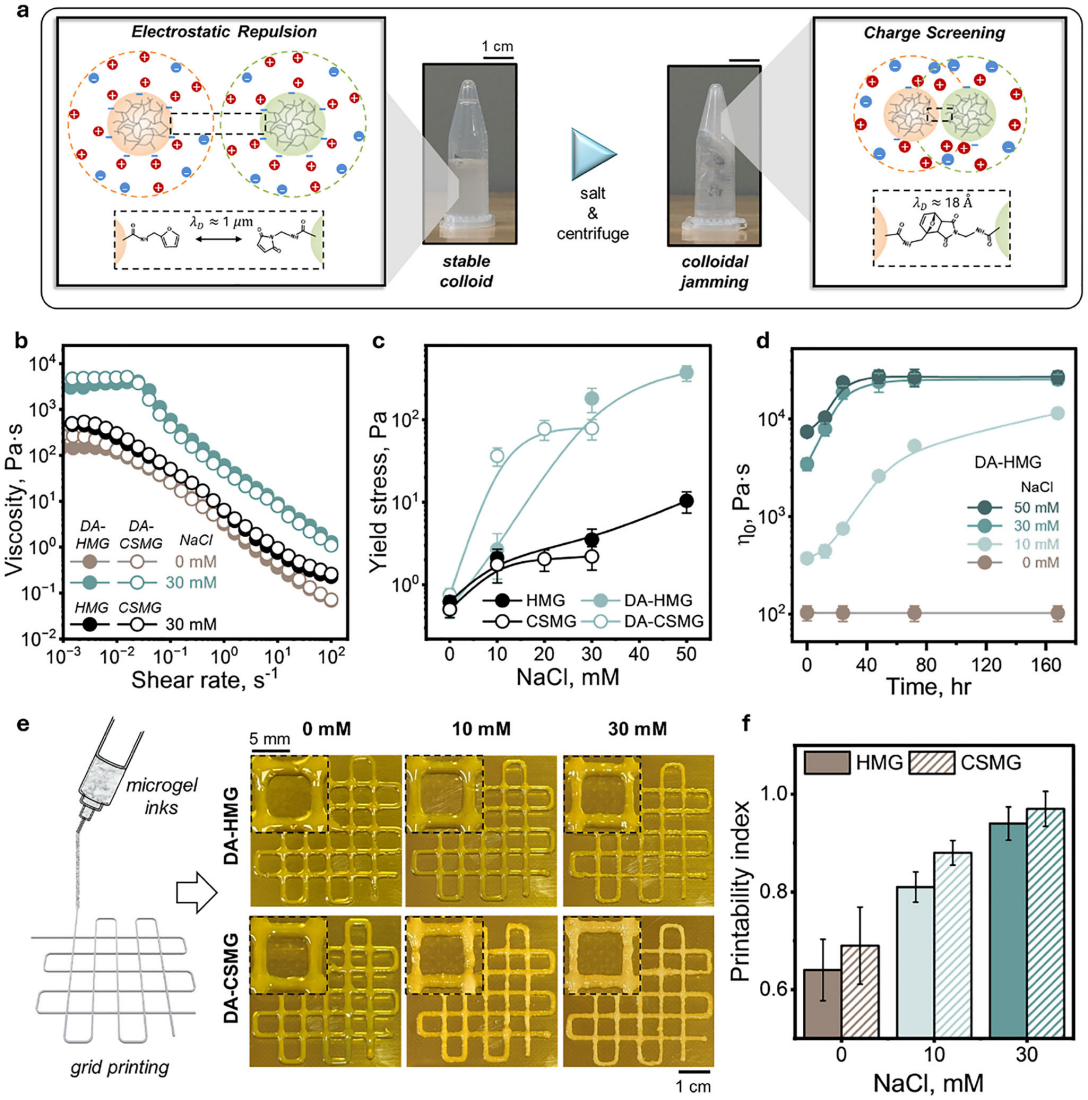
Ionic‐strength‐mediated rheological control and printability of clickable microgel inks. (a) Schematic illustration of salt‐induced DA bond formation between F‐ and M‐MGs. Electrostatic repulsion in the hydrated state hinders interparticle crosslinking (left), while charge screening upon NaCl addition and centrifugation facilitated particle jamming and bond formation (right). The scale bar in the image is 1 cm. (b) Shear‐thinning behavior of DA‐MG inks with 0 and 30 mM NaCl, measured via viscosity vs. shear rate under parallel plate geometry at 20°C. (c) Yield stress values determined from oscillatory stress sweep (1 Hz, 0.01–1000Pa, see Figure S11). (d) Time‐dependent development of zero‐shear viscosity, η_0_, extracted from the low‐shear‐rate plateau (Figure S12). (e) The printed 7 × 7 mm^2^ grid structures using DA‐MG inks at 0, 10, and 30 mM NaCl. Scale bars: 1 cm (global), 5 mm (enlarged). (f) Quantitative printability index extracted from the printed grid images.

Having established a chemically functionalized microgel toolkit that remains colloidally stable in aqueous dispersions, we next sought to convert these F‐MGs and M‐MGs into clickable microgel inks as shear‐thinning dispersions capable of forming DA‐crosslinked assemblies. Although the functional groups are reactive, freshly mixed dispersions (F‐MG + M‐MG) did not form DA‐linked assemblies in low‐salt conditions, as electrostatic repulsion prevented sufficient interparticle contact. To activate network formation, we introduced NaCl to screen surface charges and applied centrifugation to jam the microgels. This processing step transformed the initially low‐viscosity F/M‐MG dispersion into a cohesive, shear‐thinning ink (DA‐MG, Figure 3a), wherein salt‐induced compaction enabled DA bonds to form gradually under aqueous conditions.

We first quantified how salt concentration controls rheology of the inks. The salt concentrations were selected to balance effective crosslinking and colloidal stability, guided by the available carboxylic acid content in each system (0.11 M for HMG‐based and 0.08 M for CSMG‐based dispersions). The key rheological parameters, including shear‐thinning behavior, yield stress, and printability index, are critical for determining ink extrudability, shape retention, and structural stability during and after deposition [49]. Both DA‐HMG and DA‐CSMG dispersions displayed pronounced shear‐thinning behavior across all conditions (Figure 3b; S10), desirable for extrusion. Notably, increasing NaCl from 0 to 50 mM for DA‐HMG and from 0 to 30 mM for DA‐CSMG dispersions (Figure S10) significantly elevated viscosity as electrostatic screening enhanced interparticle contact upon jamming. Control experiments with unmodified HMG and CSMG dispersions (≈ 3.5 wt%, 30 mM NaCl) confirmed that DA‐MGs at matched particle concentration and ionic strength exhibited higher viscosity than their unmodified counterparts (Figure 3b), highlighting that the additional cohesion stems from interparticle DA bond formation beyond simple physical jamming. To complement viscosity results, yield stress (i.e., the minimum stress needed to initiate flow, Figure 3c) was also determined using the oscillatory stress sweeps (1 Hz, 0.01–1000 Pa, Figure S11). In all cases, DA‐MGs exhibited markedly higher yield stresses than unmodified controls at comparable salt concentrations. For example, as shown in Figure 3c, DA‐HMG reached ≈ 182 Pa compared to only ≈ 3.5 Pa for HMG, while DA‐CSMG inks displayed ≈ 79 Pa compared to ≈ 2.2 Pa for CSMG. These values also place DA‐MG inks firmly within the printable regime, where yield stress is sufficient to support continuous extrudate yet not so high as to hinder extrusion. Thus, DA bonding not only increases network connectivity but also tunes the yield stress into a range suitable for 3D printing. To minimize the influence of time‐dependent DA crosslinking, all measurements presented here were performed with the freshly prepared sample (salt addition + centrifugation), providing a consistent basis for assessing the rheological impact of ionic strength.

We also tracked the evolution of the microgel zero‐shear viscosity, η_0_, as estimated from the low‐shear‐rate plateau region in viscosity profiles (Figure S12). The increase in η_0_ over time reflects progressive interparticle crosslinking via DA bonds under aqueous conditions. For both DA‐HMG and ‐CSMG dispersions, η_0_ gradually increased and reached a limiting value within ≈ 48 hrs at NaCl concentration of 30 mM or higher (Figure 3d; Figure S13). Note that the control sample without the salt (0 mM) showed no change in η_0_ over time, suggesting that electrostatic repulsion in the absence of ionic screening prevented effective bond formation between the microgels. A direct comparison of DA‐MG inks and unmodified MG dispersions at 30 mM NaCl (Figure S14) further highlights this distinction, confirming that the observed time‐dependent viscosity reflects covalent network maturation rather than salt‐mediated physical effects. Complementary time‐resolved small‐strain rheology revealed consistent trends in storage modulus (G’), which increased monotonically over ≈ 48 h, reflecting time‐dependent increase in microgel crosslinking (Figure S15).

We next examined the impact of these rheological evolutions on printability window and actuation stability. To assess the extrusion quality of inks aged for different periods, we quantified the minimum continuous extrudate width achievable under fixed printing parameters (22 G needle, 3 mm/s, 0.6 mm layer height). Despite gradual viscosity growth, inks aged from 0–6 h produced nearly identical extrudate filaments (0.95–1.05 mm), indicating a stable pot‐life within the relevant printing timeframe (Figure S16). Only after prolonged aging (12–24 h) did extrudate filament width increase noticeably (1.15–1.45 mm), consistent with higher cohesion and micro‐cluster formation. In parallel, to further correlate rheological maturation with structural robustness, we evaluated post‐deposition stabilization by depositing simple printed elements and allowing them to rest at ambient condition for defined durations before immersion in water (Figure S17). Structures aged for <12 h showed partial or full disintegration upon immersion, consistent with insufficient DA crosslinking. After 12 h, printed elements preserved their shape upon immersion; however, these partially matured assemblies still failed during repeated pH or temperature cycling, indicating inadequate network integrity for functional actuation. In contrast, structures aged for 24 h remained intact during handling, immersion, and at least five full actuation cycles, establishing that ≈ 24 h of ambient curing is required to achieve mechanical stability during actuation. Notably, this transition coincides with kPa‐scale mechanical strength in the assemblies (tensile modulus 5–7 kPa and tensile strength 1.5–2.3 kPa; Figure S18 and Table S2). While the modulus of our system (5–7 kPa) is orders of magnitude lower than those of tough double‐network 4D printed hydrogels (typically ≈ 0.1–10 MPa) [16, 25], it is comparable to values reported for granular hydrogels used in 3D printing [22, 23] where shape stability and cohesiveness are the dominant performance criteria rather than load‐bearing capability. Together, these observations directly link the rheological maturation (η_0_ and G’ growth) to the mechanical integrity required for handling and subsequent actuation experiments. While DA‐MG inks remain printable for several hours after preparation due to their shear‐thinning nature, the DA network requires at least ≈ 24 h of unobstructed ambient aging to assure formation of a continuous DA crosslinking network for mechanical stability.   Thus, for 4D‐printing applications where repeated actuation is required, an aging period of approximately one day represents a robust and reproducible time‐to‐use guideline for both DA‐HMG and DA‐CSMG inks.

To quantify the printability of the clickable microgel inks, we employed an extrusion‐based 3D printer modified with a syringe extruder [50] to deposit a 7 mm x 7 mm grid pattern using inks containing 0, 10, or 30 mM NaCl (Figure 3e). No additives or rheology modifiers were used in any formulation, emphasizing that printability was governed solely by salt‐induced jamming and subsequent DA bond formation. Printing was performed using freshly prepared inks within 6 hrs. The printability index was calculated based on the squared pore geometry calculated as *L* ^2^/16*A*, where *L* is the side length and *A* the pore area (Figure 3f) [49]. At 0 mM NaCl, the printed structures exhibited collapsed, poorly defined pores due to insufficient cohesion. With 10 mM salt, a modest improvement was observed, with slightly better pore definition for DA‐CSMG. Finally, a higher salt concentration of 30 mM NaCl yielded well‐defined square pores for both types of inks. While 50 mM NaCl further increased yield stress, it also caused premature micro‐aggregation and reduced filament uniformity (Figure S19), making 30 mM the most reliable formulation for constructing complex 3D architectures because it provides yield stresses in the printable regime (DA‐HMG: 182 Pa and DA‐CSMG: 79 Pa; Figure 3c), maximizes filament fidelity in grid tests (Figure 3e,f), and affords a practical working time window for inks (Figure S16).

### 2.3 4D Printing of Freeform Microgel‐Based Constructs

**FIGURE 4:**
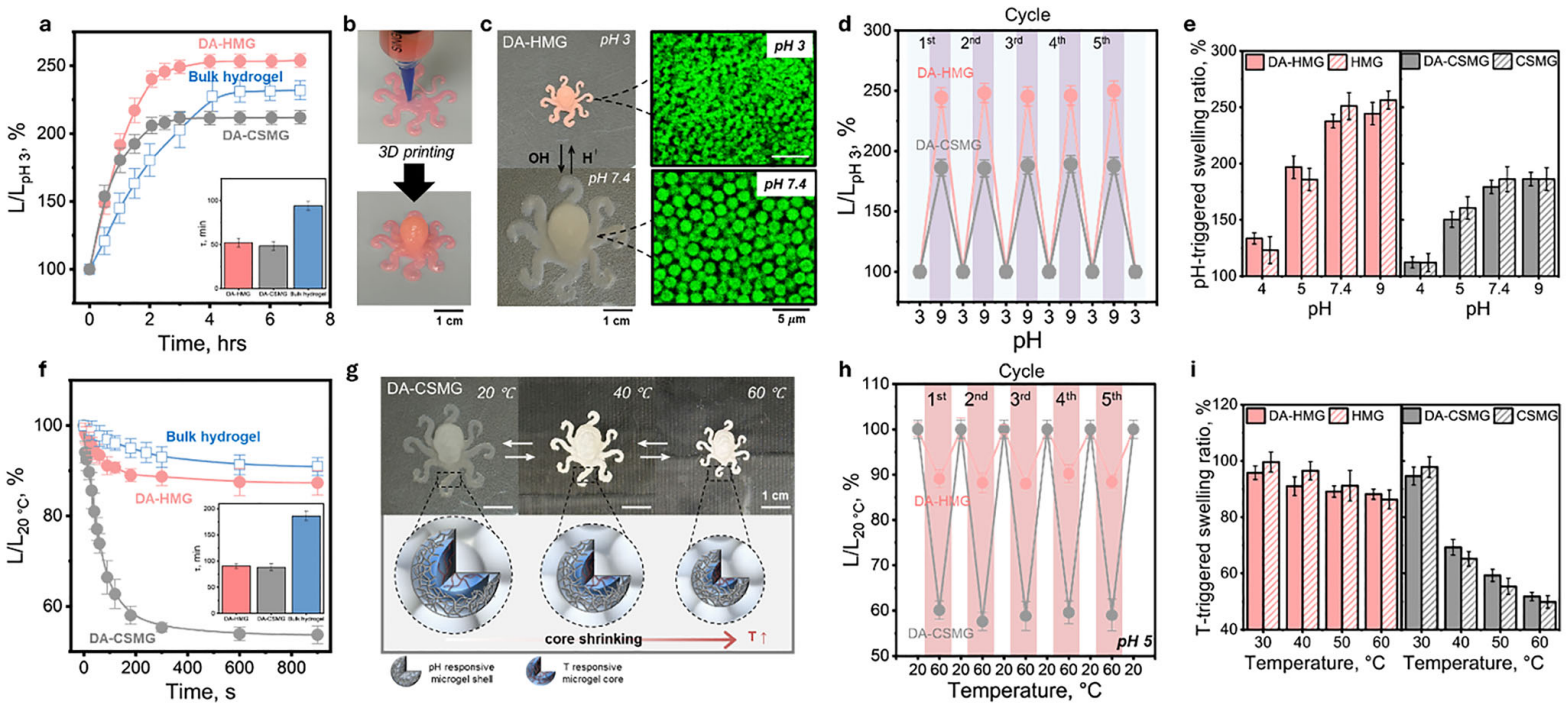
Multi‐stimuli response of 3D printed DA microgel assemblies. (a) comparison of pH‐triggered swelling kinetics of DA‐microgel assemblies and composition‐matched bulk P(NIPAM‐co‐AAc) hydrogels (50:50 mol%, 2 wt.%), measured using identically the molded star‐shaped samples (Figure S20). All samples were pre‐equilibrated at pH 3 and transferred to pH 9. (b) Octopus‐shaped structures printed from DA‐HMG ink (dyed with Rhodamine 6G for visual contrast) at 30 mM NaCl. The scale bar is 1 cm. (c) pH‐triggered shape change of the printed assemblies (Scale bar: 1 cm) at pH 3 and 7.4, along with confocal microscopy images (scale bar 5 µm) illustrating pH‐induced size changes of individual HMG particles within the printed assemblies at each pH; the samples are stained with fluorescein and rhodamine 6G dyes, respectively. (d) Reversibility of pH‐responsive behavior across multiple cycles. (e) pH‐triggered swelling ratio of printed octopus DA‐MG assemblies (L/L_pH3_), compared to the corresponding swelling ratio of individual microgel particles (D/D_pH3_). (f) Temperature‐triggered response kinetics of DA‐CSMG assemblies compared with composition‐matched bulk hydrogels (50:50 mol%, 2 wt.%). The samples were heated from 20°C to 60°C. (g) Temperature‐triggered contraction of a 3D‐printed DA‐CSMG object (octopus) upon heating from 20°C to 60°C; schematics illustrate shrinking of the temperature responsive core. The scale bar is 1 cm. (h) Reversibility of temperature responsive behavior across multiple cycles. (i) Temperature‐triggered swelling ratio of the printed DA‐MG octopus (L/L_20°C_), shown alongside the swelling ratio of the corresponding individual microgel particles (D/D_20°C_) at pH 5.

Based on the rheological design rule, and maturation behavior of the clickable microgel inks, we next examined whether the macroscale properties of printed structures directly inherit the programmed responsiveness of the individual microgel building blocks. To quantitively compare actuation performance with conventional hydrogels, we first evaluated response kinetics using molded star‐shaped samples (Figure S20) for both DA‐MG assemblies and composition‐matched P(NIPAM‐co‐AAc) bulk hydrogels (50:50 mol%, 2 wt.%). The use of star geometries ensured identical sample dimensions, allowing direct comparison despite the fact that bulk hydrogels cannot be printed. As shown in Figure 4a, upon increasing the pH from 3 to 9, DA‐MG assemblies displayed a more rapid swelling transition (τ ≈ 50 min) compared to the bulk hydrogel control (τ ≈ 90 min). This enhancement is likely due to the intrinsic porosity of the microgel‐based hydrogel network, which facilitates rapid solvent uptake and ion diffusion—an advantage widely recognized in granular hydrogel systems [27, 31, 32].

Guided by these findings, DA‐MG inks formulated at 30 mM NaCl (previously shown to balance cohesion and extrudability) were used for DIW of octopus‐shaped objects (Figure 4b). After 24 h of ambient curing (established above as the minimum maturation time for cycle‐stable cohesive networks), the printed structures retained their shape and withstood handling without fragmentation. When subjected to a pH change (3 → 7.4), the constructs exhibited reversible dimensional changes (Figure 4c; Figure S21), while the contraction and expansion of the constituent microgels were visualized through changes in morphology of the assembled particles, as confirmed by confocal microscopy. The constructs preserved their shape and performance across five pH cycles with no cracking or delamination (Figure 4d). Response of the swelling ratios of the octopus (L/L_pH3_, determined by inscribing its largest dimension in a circle and taking the circle diameter as L) closely matched those of the original HMG and CSMG particles (D/D_pH3_) characterized independently by fluorescence microscope, confirming the direct transfer of microscale responsiveness into macroscale actuation (Figure 4e). This direct correspondence demonstrates the modular programmability of the assembled structures, with the bulk‐level swelling precisely inheriting the responsiveness of their microgel building blocks.

We then investigated thermally induced actuation. The DA‐CSMG assemblies contracted rapidly as temperature increased from 20°C to 60 °C (Figure 4g), with a characteristic response time τ ≈ 80 s, approximately two‐fold faster than a bulk hydrogel of identical chemistry (τ ≈ 180 s) (Figure 4f). In contrast, DA‐HMG showed insignificant thermal response under the same conditions (Figure S22 for a set of images), in agreement with the suppressed LCST behavior of P(NIPAM‐co‐AAc) at pH 5. DA‐CSMG structures also demonstrated reversible multiple heating‐cooling cycles between 20°C and 60°C without loss of cohesion (Figure 4h), highlighting the robustness of the chosen click chemistry for interparticle crosslinking under repeated thermal actuation. In addition to cyclic actuation stability within the temperature range between 20°C and 60°C, the printed DA‐CSMG constructs also exhibited structural integrity during prolonged exposure to elevated temperatures. When immersed in water at ≈ 90°C for 24 h (i.e., near the onset of the rDA endotherm) the structures retained integrity, indicating that DA linkages maintained network connectivity well below the rDA peak temperature (≈120°C). Although 24 h exposure to ≈ 90°C induced irreversible shrinkage due to network reconfiguration, the constructs retained responsiveness to external stimuli, albeit with reduced amplitude (Figure S23). The amplitude of changes in swelling ratio in response to temperature (L/L_20°C_) again aligned closely with the thermoresponsive behavior of individual microgels (D/D_20°C_), confirming that the macroscopic behavior of DA‐MG assemblies is predictably programmed by the constituent microgels (Figure 4i). Together, these results demonstrate how rational design of clickable microgel inks, guided by understanding and tailoring of ink rheology, enables the direct construction of multi‐responsive structures with high shape fidelity. The seamless transfer of the particle‐level functionality into the responsiveness of the macroscale printed objects demonstrates the versatility of this modular assembly approach.

### 2.4 Programming Localized Responses for Stimuli‐Triggered Shape Morphing

By leveraging the printability and multi‐stimuli responsiveness of our DA‐MG inks, we next explored spatially resolved patterning to achieve differential stimulus‐guided shape morphing. While both HMG and CSMG particles respond to pH and temperature, their magnitudes of response differ substantially: HMG exhibits larger pH‐driven swelling, whereas CSMG undergoes far greater thermally induced contraction. This differential responsivity provides a mechanism for localized actuation, even in the presence of modest crosstalk.

The spontaneous, self‐adhesive nature of our clickable microgel inks enables seamless integration of different microgel components into continuous, mechanically robust constructs. To determine the appropriate healing time for producing reliable HMG/CSMG interfaces, we leveraged the quantitative self‐healing kinetics established in Figure S24, which show that DA‐MG strips recover most of their mechanical strength after 6 h of interfacial contact. Based on this recovery profile, all multimaterial samples were assembled and allowed to heal for 24 h prior to mechanical testing. Under these conditions, interfacial adhesion strength (≈ 2.1 kPa) approached the tensile strength of the individual DA‐MG networks (Table S2), confirming robust interdomain cohesion.

Dual‐color fluorescence imaging of labeled HMG (AZDye 488) and CSMG (AZDye 594) domains (Figure S25) further reveals sharply preserved interfaces without drift or intermixing during network maturation, demonstrating that DA‐mediated particle‐particle bonding achieves both mechanical integration and spatial fidelity.

**FIGURE 5:**
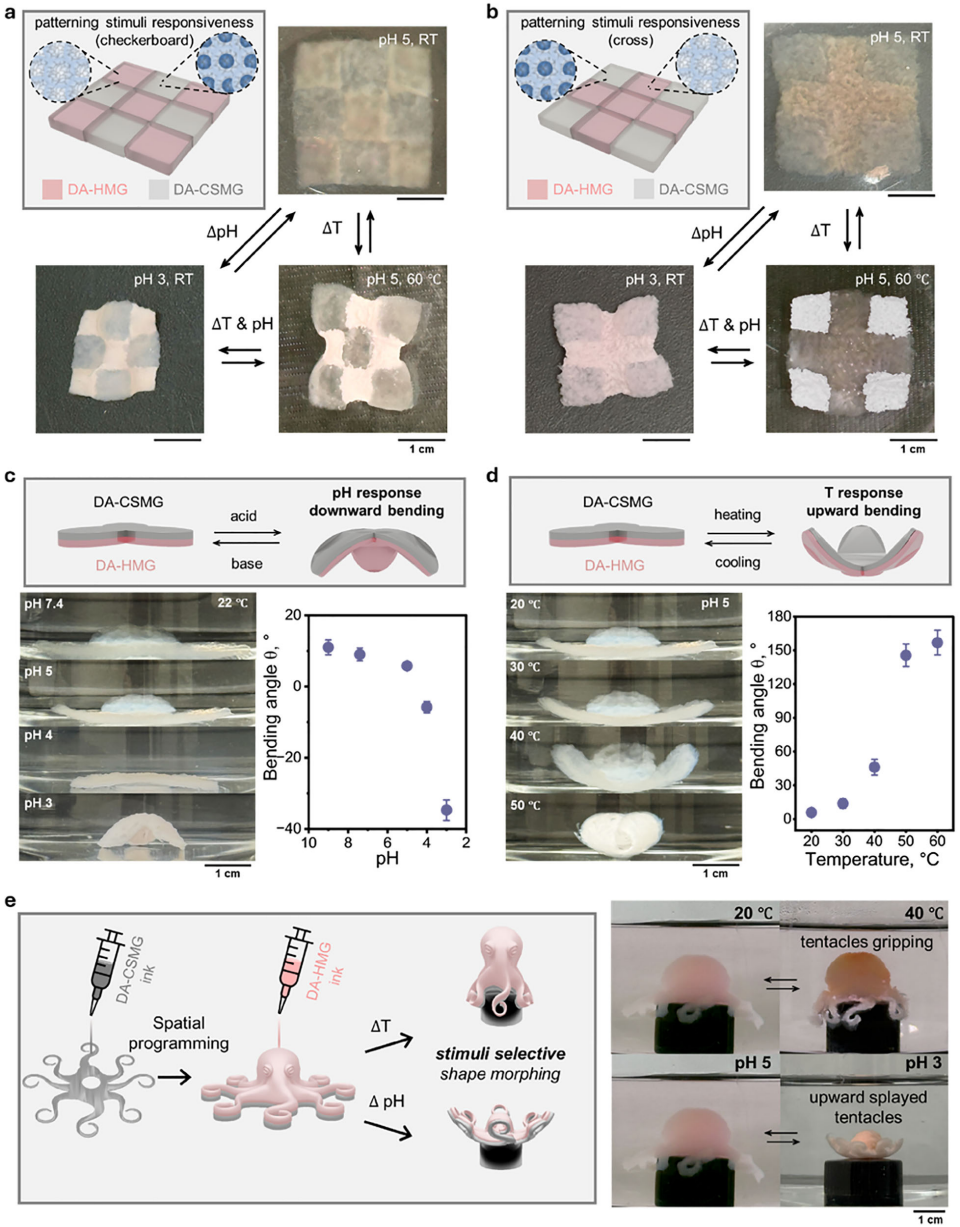
Spatially programmed, differential shape morphing enabled by multi‐material DA‐MG assemblies. (a) checkerboard and (b) cross‐patterned planar constructs printed with alternating DA‐HMG and DA‐CSMG domains. (c) pH‐driven direction‐controlled bending of bilayer petal actuator composed of DA‐HMGs and DA‐CSMGs layers. (d) Temperature‐driven bending of the same bilayer actuator. (e) Octopus‐shaped soft actuators fabricated using multiple microgel inks: bottom tentacles are printed with DA‐CSMGs and top tentacles/body with DA‐HMGs. Selective downward gripping and upward splayed tentacles were triggered by temperature and pH changes, respectively. All scale bars are 1 cm.

To visualize spatially encoded responses, we first printed the checkerboard and cross‐patterned planar assemblies composed of alternating HMG and CSMG domains (Figure 5a,b). Upon exposure to either acidic/basic environment or heat, the domains swelled to different extents (2.5 × vs. 1.9 × for the pH shift; 10 % vs. 50 % for the temperature change), resulting in spatio‐selective shape morphing driven by differential swelling.

To more directly quantify this differential actuation, we fabricated bilayer “3 petal” geometries composed of different microgel components (Figure 5c,d). For pH‐triggered actuation (from pH 3 to 9), the mismatch in expansion drove the petals to bend toward the HMG side, generating a bending angle change of ≈ 50^○^. Conversely, under thermal stimulation (20 → 60°C), the substantially larger contraction of the CSMG layer reversed the bending direction, producing a much larger bending angle change of ≈ 150^○^. These opposite, stimulus‐specific bending directions highlight that the deformation pathway is governed by the relative magnitude of each domain's response, enabling directional actuation despite modest pH‐temperature crosstalk.

Finally, heterogenous multi‐material 3D constructs, such as octopus‐shaped objects, were printed with DA‐CSMG in the bottom tentacles and DA‐HMG in the upper tentacles and body (Figure 5e). Heating induced selective downward curling to the CSMG domains, while pH shift produced upward splaying motion to the HMG domains, illustrating spatially programmable actuation. Taken together, these results highlight key advantages of clickable‐microgel constructs over conventional hydrogel systems, such as the ability to locally encode different material responses via direct writing, spontaneous shape stabilization without the need for post‐processing, strong multimaterial binding, and spatioselective shape morphing.

## 3 Conclusions

While recent advances in hydrogels have enabled spatial encoding of multiple stimuli‐responses through droplet‐based template [39], reversible adhesive interfaces [8], or 3D printing with customized chemistries [11], these systems are constrained by scalability, simple geometries, and complex formulation and processing steps, respectively. Our approach overcomes these limitations, offering potentially a universal bottom‐up strategy for encoding versatile, localized responses in soft materials. The key novelty of our approach lies in the development of responsive microgel inks by encoding distinct stimuli responses into individual microgels prior to assembly. We created clickable inks that are self‐adhesive, additive‐free, and printable directly under ambient conditions, turning a “ready‐to‐use” responsive gel into an autonomously maturing ink system. This enabled the assembly of complex structures with precisely localized, differential swelling, without requiring photoinitiators, UV‐curing, or rheology modifiers. The fidelity of printed structures was preserved during environmental changes, and the assemblies responded in a programmable, locally specific manner that is consistent with the responsiveness characteristics of the microgel building blocks. We demonstrated the potential of this platform through multimodal actuation scenarios: patterned assemblies exhibiting domain‐specific morphing, bilayer structures with direction‐controlled bending, and octopus‐like structures performing selective gripping motions under pH or temperature stimuli —all driven by the spatially encoded responses. In all cases, the macro‐scale behavior repeated the responsiveness of the constituent microgels, suggesting predictability of the materials functionality due to the facile transfer of the individual particle design to the material's collective behavior.

Several directions remain open for refinement. While relatively slow ‘clicking’ between microgels dispersed in a liquid medium can be advantageous for preserving ink stability during handling and printing, the kinetics can be tuned as a design variable. For applications where faster interparticle coupling is preferred, strategies such as surface‐localized presentation of reactive groups (i.e., surface tethers) or the use of faster click reactions (e.g., the inverse electron demand DA cycloaddition [51]) could be implemented to trigger rapid bonding on demand. Moreover, future expansion to orthogonal dynamic chemistry (e.g., photo‐switchable or redox‐responsive) [52] would further broaden the range of stimuli that can be independently encoded and patterned within the same constructs. In parallel, incorporating anisotropic patterns or spatial compositional gradients offers routes toward directional shape morphing, accelerating the transition of this platform into functional soft robotics, biomedical, or adaptive material systems [15].

## 4 Experimental Section

### 4.1 Materials

N‐Isopropylacrylamide (NIPAM, ≥99.0%), acrylic acid (AAc, ≥99.0%), N,N′‐methylenebisacrylamide (Bis, ≥99.0%), potassium persulfate (KPS, ≥99.5%), furfuryl amine (≥99%), N,N,N′,N′‐tetramethylethylenediamine (TEMED, ≥99.5%), sodium chloride (NaCl, ≥99.5%), fluorescein sodium salt, and Rhodamine 6G were all purchased from Sigma‐Aldrich and used as received. 1‐(2‐Aminoethyl)maleimide (hydrochloride salt, ≥93.0%) and 4‐(4,6‐dimethoxy‐1,3,5‐triazin‐2‐yl)‐4‐methylmorpholinium chloride (DMTMM, ≥95.0%) were purchased from TCI Chemicals and used without further purification. Buffer solutions were prepared using standard analytical‐grade reagents: citric acid/sodium citrate (for pH 3–5), sodium phosphate monobasic/dibasic (for pH 6–7.4), and Tris(hydroxymethyl)aminomethane (TRIS) for pH 9. All solutions were made in deionized (DI) water with resistivity >18.2 MΩ·cm.

### 4.2 Synthesis of HMG and CSMG

HMG and CSMG were synthesized via surfactant‐free precipitation polymerization. The monomer feed for HMG consisted of 4 mmol NIPAM, 4 mmol AAc, and 0.16 mmol Bis in 100 mL deionized (DI) water. After deoxygenation by vacuum and Ar purging, the mixture was heated to 60°C with stirring for 15 min. Polymerization was initiated by injecting 0.2 mmol KPS dissolved in 2 mL DI water. After 8 min, once turbidity appeared, a pre‐mixed solution containing 7 mmol NIPAM, 7 mmol AAc, and 0.28 mmol Bis in 6 mL DI water was fed at 9 mL/h using a syringe pump. CSMGs were synthesized using a similar procedure. The monomer feed solution for core was composed of 8 mmol NIPAM in 100 mL DI water, initiated as above. For shell formation, the same NIPAM:AAc:Bis ratio was used in the second feed solution and injected under identical conditions.

### 4.3 Microgel Functionalization with Diels‐Alder Reactive Groups

Furan and maleimide groups were introduced via amide coupling using DMTMM chemistry. Furfuryl amine or 1‐(2‐aminoethyl)maleimide was reacted with the carboxylic acid groups on HMG or CSMG at varying molar ratios (0.005–0.2 mmol reagent per mL of 1.7 wt.% microgel dispersion) in a 1:1 (v/v) ethanol/pH 5 MES buffer solution. For experimental consistency, 0.03 mmol of reagents was used for most studies, corresponding to molar ratios of 1:3.7 (HMG) and 1:2.7 (CSMG) relative to the carboxyl content determined by titration. Reactions proceeded overnight at room temperature. After overnight coupling, dispersions were purified by centrifugation‐resuspension (× 5 cycles): samples were centrifuged, the supernatant was discarded, and the pellet was resuspended in DI water to remove residual solvent and salts. Functionalization was confirmed by ATR‐FTIR (see Figure S5), showing characteristic furan and maleimide peaks. Estimated coupling efficiencies under the conditions are summarized in Figure S6.

### 4.4 Preparation of Clickable Microgel Inks

To prepare DA‐crosslinkable inks, equal volumes of furan‐modified (F‐MG) and maleimide‐modified (M‐MG) microgels were mixed at identical particle concentrations (1.7 wt.%). Although the functionalized microgels (“toolkit components”) remain colloidally stable for months in aqueous dispersion, DA bond formation does not occur appreciably without forced contact. Therefore, inks were prepared by salt + centrifugation‐assisted jamming.

### 4.5 Characterization

Confocal microscopy was performed using a LEICA SP8 with 100x/1.4 NA oil immersion lens. Fluorescein sodium salt was used at pH 9 to visualize microgel internal structures based on electrostatic contrast. pH‐responsive diameter changes were imaged using a Zeiss Axio Vert.A1 fluorescence microscope (40x/0.65 NA) with fluorescein (for pH 3 and 4) or Rhodamine 6G (for pH 5–9) depending on pH. For imaging, 0.2 wt.% microgel dispersions were dyed with 0.2 mg/mL dye solution and immobilized on branched polyethylenimine‐coated glass. >30 particles were analyzed per condition.

DLS (Malvern, Zetasizer Nano ZS) was used to monitor temperature‐responsive size changes of microgels (0.01 wt%, pH 5 citrate buffer).

An Asylum Research Cypher Atomic Force Microscope (AFM) was used to collect scanning probe micrographs and measure topography. The Si cantilever used was RFESPA‐75 (Bruker, USA) with 3 N/m nominal spring constant, 75 kHz nominal resonance frequency, and 8 nm nominal tip radius. Tapping mode scans were performed using a 150 mV setpoint and 0.50 Hz scanning speed.

Rheological measurements were performed on a DHR‐2 rheometer (TA Instruments) with 40 mm parallel plate and 100 µm gap at 20°C. Dispersions were jammed by centrifugation at 8170 rcf and 20°C prior to measurements. Flow ramps (0.001–100 s^−^^1^) and stress sweeps (1 Hz, 0.01–1000 Pa) were used to assess salt‐induced change in viscosity and yield stress while time‐sweep measurements were performed to track average storage modulus (G’) during DA‐mediated network maturation.

### 4.6 Filament Stability (Ink Pot‐Life)

Filaments were printed at fixed parameters (22G nozzle, 3 mm/s, 0.6 mm layer height). Minimum continuous filament width was measured at ink ages of 0, 1, 2, 6, 12, and 24 h (Figure S16). Width stability within ≤ 6 h defines the practical pot‐life of the ink.

### 4.7 Post‐Curing / Time‐to‐Use Stability Test

Simple elements were extruded and allowed to rest under humidified ambient conditions for varying times (0, 1, 2, 6, 12, 24 h). After aging, they were immersed in water and subjected to cyclic pH or temperature actuation (Figure S17). Aging ≥ 24 h produced structures that survived ≥ 5 actuation cycles and resisted fragmentation.

### 4.8 3D Printing of Microgel Assemblies

DA‐MG inks were printed using a modified Flashforge Finder 3 with a syringe‐based extrusion system (22G tip). The motion control was repurposed to actuate syringe plungers. Print parameters: 3 mm/s speed, 0.6 mm layer height, concentric infill. Salt concentrations: 30 mM for both HMG and CSMG. Only DA‐HMG dispersion was dyed (5 µL of 0.2 mg/mL Rhodamine 6G per 1 mL) for visual contrast, centrifuged to jam, and used within 6 h. All printed structures were incubated in sealed containers at 4°C for 24 h to allow crosslinking while preventing drying.

For the swelling kinetics comparison with the bulk hydrogel, star‐shaped silicone molds were used to cast both DA‐MG and bulk hydrogel samples (2 wt.%). DA‐MGs were prepared with 30 mM NaCl and incubated at 4°C for 24 h. Bulk hydrogels were synthesized by mixing the same monomer composition (1 mmol of NIPAM and AAc with 0.04 mmol of Bis crosslinker) in 4 mL DI water, initiated by adding 0.1 mmol of KPS, followed by 15 µL TEMED directly into the mold. After overnight polymerization and three‐times DI water washes, all samples were pre‐shrunk in pH 3 buffer overnight. Swelling was monitored upon transfer to pH 9 by measuring linear expansion using image analysis.

### 4.9 Mechanical Testing

Molded strips (6 mm × 20 mm × 1 mm) of DA‐HMG, DA‐CSMG, and healed HMG/CSMG interfaces were tested on a TA DMA 850 at a strain rate of 0.01 s^−1^.

### 4.10 Stimuli‐Responsive Swelling

pH responsiveness was tested by immersing printed assemblies in buffered solutions (pH 3–5: citrate; pH 7.4: phosphate; pH 9: TRIS), equilibrated ≥2 h per step. Temperature‐responsive swelling was tested in pH 5 citrate buffer with pre‐heated baths (30–60°C). Swelling was quantified by measuring diameters from circle fits in ImageJ.

### 4.11 Dual‐Color Confocal Imaging of Multimaterial Interfaces

To visualize interfacial integrity between domains, HMG and CSMG particles were separately labeled with AZDye 488 Cadaverine and AZDye 594 Cadaverine (0.1 mol%), respectively, via DMTMM coupling. Each labeled F‐MG was mixed with unlabeled M‐MG and processed into inks. Layered (bilayer) assemblies were printed and matured in ambient air for 24 h. Cross‐sections were imaged using a Zeiss Axio Vert.A1 microscope with 5 × objective, collecting channels separately using emission filters centered at 520 nm for AZDye 488 and 610 nm for AZDye 594. The two channels were subsequently overlaid using ImageJ to visualize domain boundaries and interfacial integrity.

## Conflicts of Interest

The authors declare no conflict of interest.

## Supporting information




**Supporting File**: advs74188‐sup‐0001‐SuppMat.docx.

## Data Availability

The data that support the findings of this study are available from the corresponding author upon reasonable request.
