# Supplementary material for: Clickable Microgel Inks Enable Spatioselective, Multi‐Stimuli Programmable Assembly of Materials
Source: Adv Sci (Weinh). 2026 Jan 30;13(20):e20526. doi: 10.1002/advs.202520526 (PMC13067792; doi:10.1002/advs.202520526)
Supplement: Supplementary file 1 — Supporting File: advs74188‐sup‐0001‐SuppMat.docx. [file ADVS-13-e20526-s001.docx]

**Supporting Information**

**Clickable Microgel Inks Enable Spatioselective, Multi-Stimuli Programmable Assembly of Materials**

*Junho Moon^1^, Frank Gardea^2^, Madeline A. Morales^3^, and Svetlana Sukhishvili^*1^*

^1^Department of Materials Science and Engineering, Texas A&M University, College Station, TX 77843, USA

^2^Army Research Directorate, U.S. Army Combat Capabilities Development Command Army Research Laboratory South, College Station, TX 77843, USA

^3^Army Research Directorate, U.S. Army Combat Capabilities Development Command Army Research Laboratory, Aberdeen Proving Ground, MD 21005, USA

**Supplementary Tables**

**Table S1**. Summary of representative multi-stimuli-responsive hydrogel systems, highlighting stimulus type, actuation timescales, remote addressability, and post-processing requirements.

| Hydrogel type / representative work | | Stimuli | Actuation time | Remote addressability | Need for post-processing |
| --- | --- | --- | --- | --- | --- |
| Multi-stimuli printed systems | Narupai et al., ***Adv. Funct. Mater.*** 2021^[1]^ | Temperature, pH, enzymatic degradation | - Temp: $\approx$10 min - pH: $\approx$ 1 h | X | UV curing |
|  | Arsuffi et al., ***Adv. Funct. Mater.*** 2024^[2]^ | Temperature, pH, ions | - Temp/salt: < 300 s - pH: $\approx$ 15 min |  | UV curing + Ca^2+^ ionic crosslinking |
| Droplet-templated gels (Downs et al., *Nat. Chem.* 2020)^[3]^ | | Temperature, light, magnetic field | Temp: $\approx$500 s | light addressing (photothermal effect) | UV curing, oil removal, droplet templating steps |
| Reversible adhesive assembled hydrogel (Liu et al., *Nat. Chem.* 2024)^[4]^ | | Temperature, pH, magnetic field | Not reported | X | heat/UV light trigger used to activate adhesive |
| Granular hydrogels | | To our knowledge, no previously reported granular hydrogel system exhibits intrinsic responsive actuation to multiple environmental stimuli | | | |
| This work | | Temperature, pH | - Temp: $\approx$80 s - pH: $\approx$ 50 min | X | None (no heating, UV, or chemical triggers needed) |

**Table S2.** Summary of tensile strength ($\sigma_{max}$), elongation at break ($\varepsilon_{b})$, tensile modulus (*E*), interfacial adhesion strength ($\sigma_{interface}$) of self-healed DA-HMG/DA-CSMG interface, and small-strain shear modulus (G’). Error bars represent standard deviations (n = 5).

|  | $\sigma_{max}$ (kPa) | $\varepsilon_{b}$ (%) | *E* (kPa) | $\sigma_{interface}$ (kPa) | G’ (kPa) |
| --- | --- | --- | --- | --- | --- |
| DA-HMG | 2.26 $\pm$ 0.38 | 32.2 $\pm$ 5.2 | 7.06 $\pm$ 0.41 | 2.11 $\pm$ 0.28 | 0.76 $\pm$ 0.14 |
| DA-CSMG | 1.65 $\pm$ 0.62 | 35.6 $\pm$ 8.2 | 5.29 $\pm$ 0.58 |  | 0.69 $\pm$ 0.12 |

**Supplementary Figures**


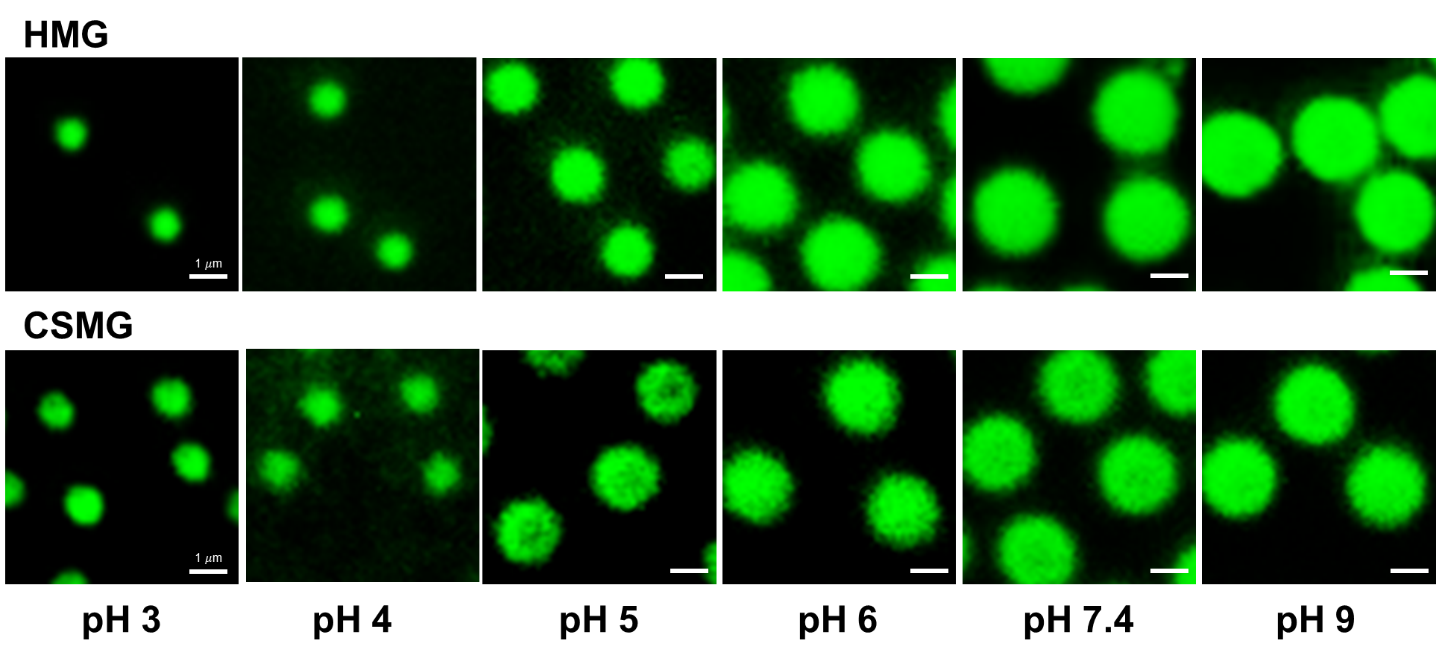


**Figure S1**. Fluorescence microscopy images of homogeneous microgels (HMG) and core–shell microgels (CSMG) at different pH conditions. Representative images of HMG (top panel) and CSMG (bottom panel) dispersions taken at pH 3, 4, 5, 6, 7.4, and 9, showing their pH-responsive swelling behavior. At acidic conditions (pH 3 and 4), fluorescein sodium salt interacts with protonated carboxyl groups in microgels through hydrogen bonding, resulting in uniform fluorescence. At neutral to basic pH (pH 5 to 9), Rhodamine 6G preferentially associates with the deprotonated microgels, enabling visualization of particles. Images were acquired using fluorescence microscopy (40x objective) and demonstrate that both HMG and CSMG expand in size with increasing pH due to the ionization of carboxylic groups.


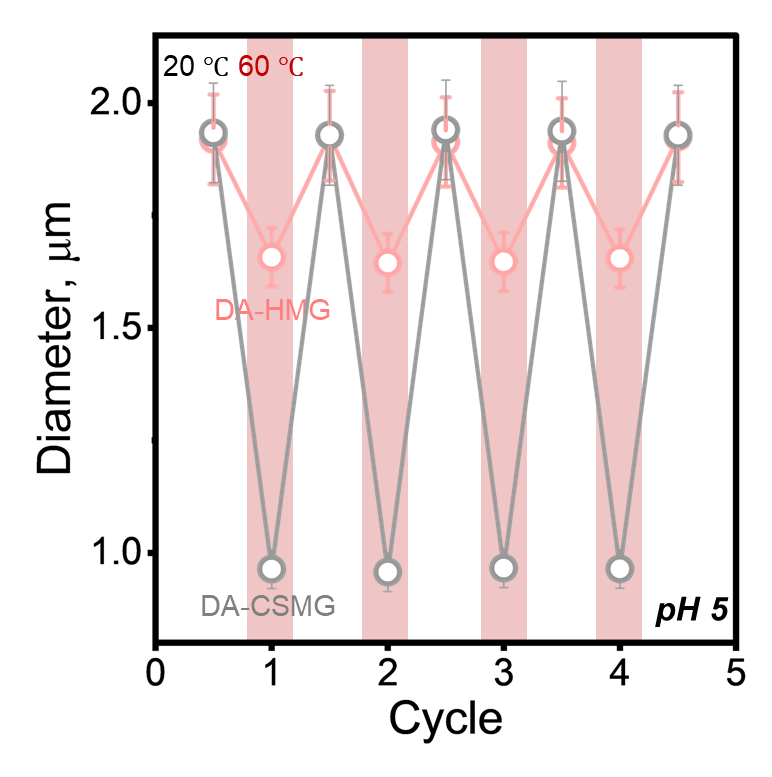


**Figure S2**. Reversible temperature responsive behavior of DA-MGs over multiple heating–cooling cycles. Dynamic light scattering (DLS) measurements of microgel hydrodynamic diameters during five consecutive temperature cycles (20 ℃ to 60 ℃) in pH 5 citrate buffer. Both DA-HMG and DA-CSMG exhibited reversible size changes with minimal hysteresis, confirming stable thermo-responsiveness under repeated cycling. The more pronounced contraction observed in DA-CSMG is attributed to the PNIPAM-based core shrinkage, while DA-HMG shows minimal temperature sensitivity at pH 5 due to the suppressed LCST behavior of the P(NIPAM-co-AAc) network.

**
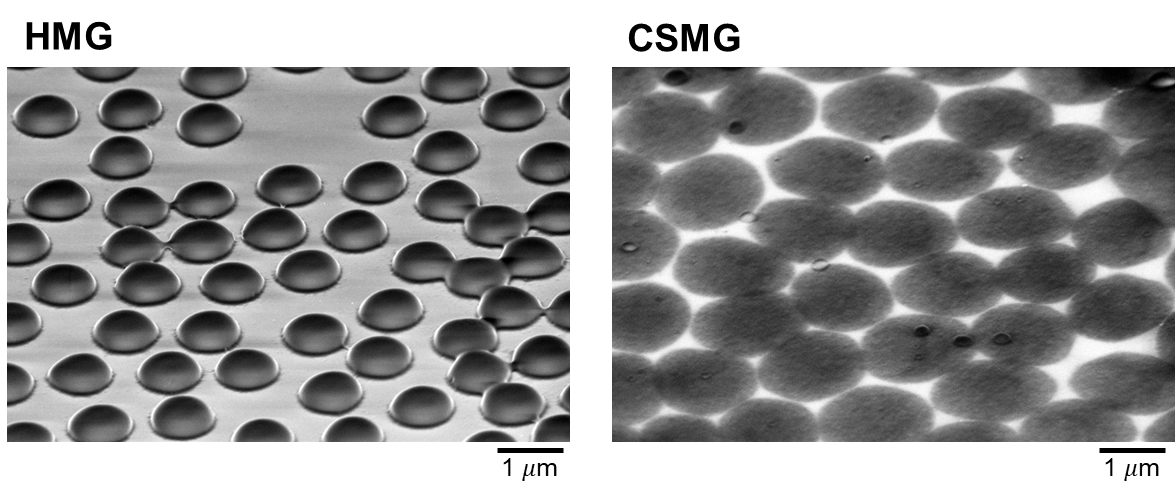
**

|  | wet | dry |
| --- | --- | --- |
| HMG | 0.9 µm | 0.95 µm |
| CSMG | 1.1 µm | 1.9 µm |

**Figure S3.** SEM images of dried HMG and CSMG deposited from pH 3 dispersions. HMG and CSMG dispersions prepared at pH 3 were drop-cast onto silicon wafers and dried under ambient conditions prior to imaging. A ThermoFisher Helios G4 FIB-SEM was used to observe the surface morphology of the dried microgels. SEM images were acquired at 1 keV and 21 pA with a 50 ns dwell time using a through-lens detector (TLD) for secondary electron imaging. All images were captured at a 50° incidence angle to enhance surface feature contrast. The accompanying table compares the average diameters in the hydrated state (from fluorescence microscopy at pH 3) and the dried state (from SEM), highlighting the dimensional change upon drying.


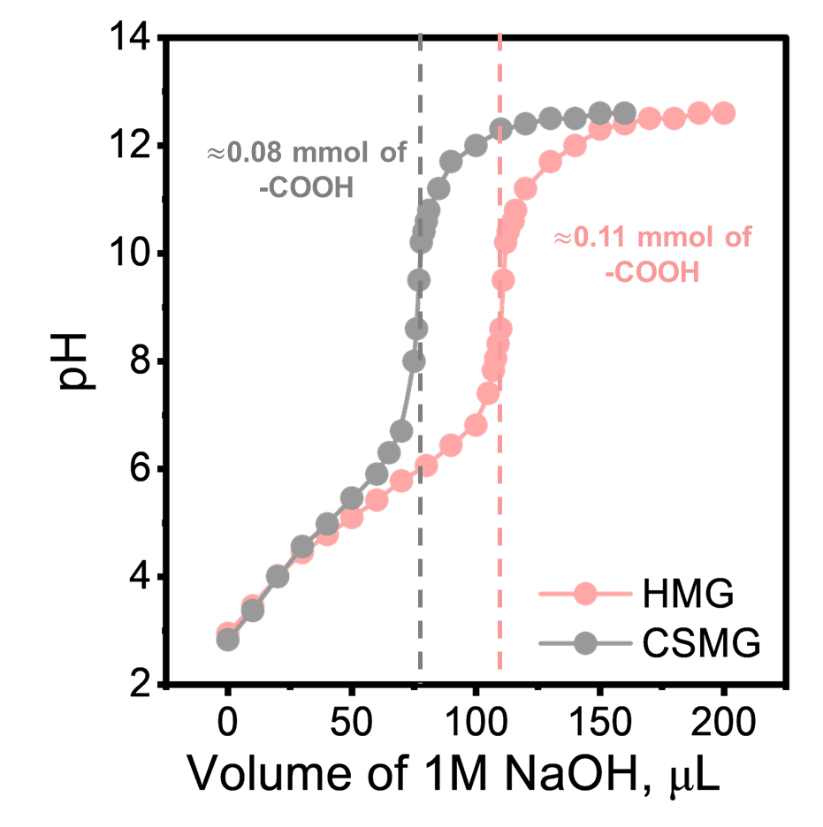


**Figure S4.** Potentiometric titration curves used for determining the amount of carboxylic groups in HMG and CSMG microgel dispersions. pH was monitored during the direct titration of 1 mL of ~1.7 wt% microgel dispersion with 1 M NaOH. The volume of NaOH added at the equivalence point corresponds to the amount of titratable carboxylic groups in each dispersion. The content of carboxylic groups was determined to be 0.11 and 0.08 mmol/mL for 1.7 wt% HMG and CSMG dispersions, respectively. Dashed lines indicate the equivalence points.

**
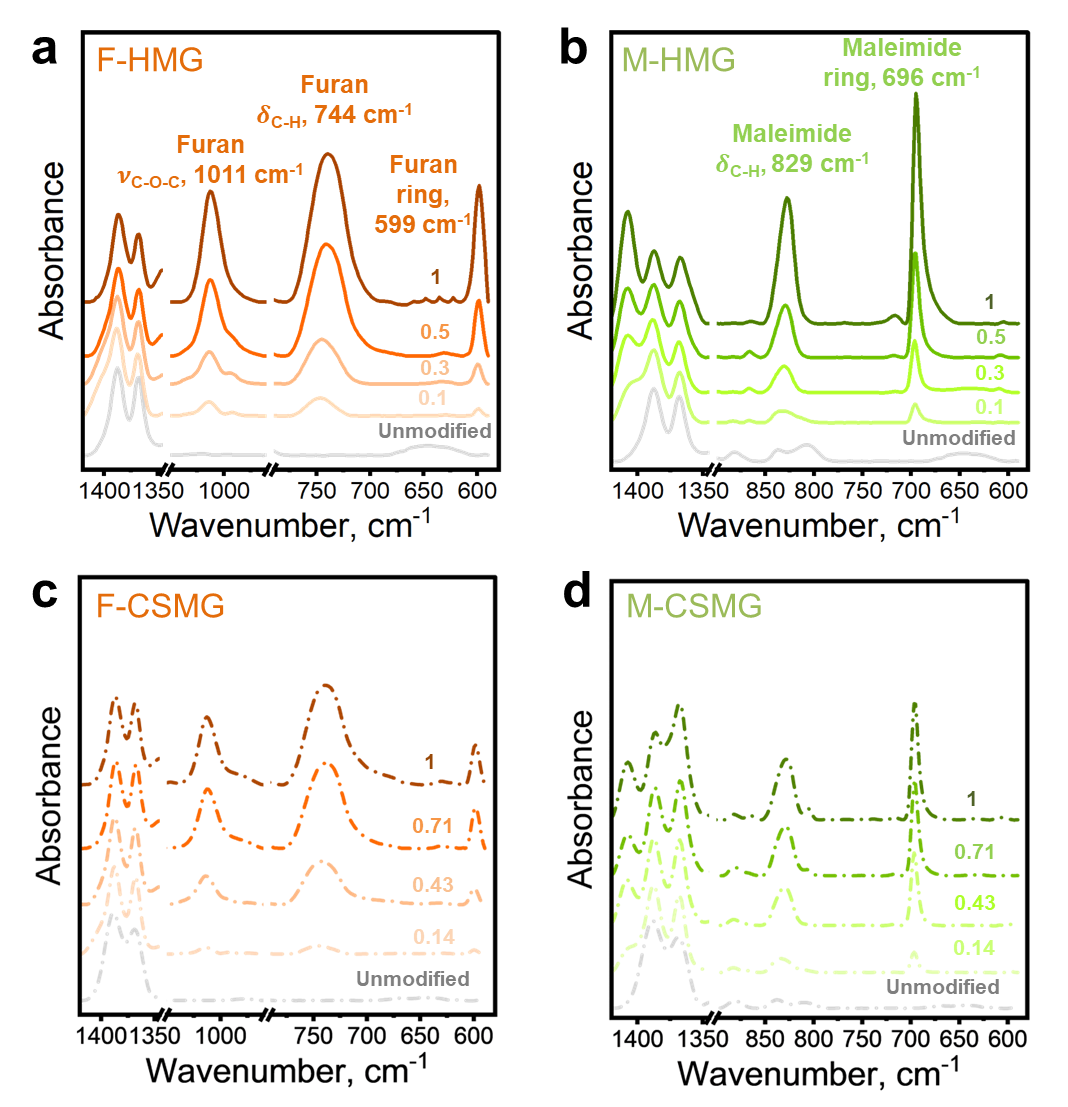
**

**Figure S5**. FTIR spectra confirming furan and maleimide functionalization of HMG and CSMG. (a) FTIR spectra of furan-functionalized HMG (F-HMG), showing characteristic peaks at 1011 cm^-1^ (C–O–C stretching), 744 cm^-1^ (C–H bending), and 599 cm^-1^ (furan ring bending)^[5]^. (b) FTIR spectra of maleimide-functionalized HMG (M-HMG), with the peaks at 829 cm^-1^ (C–H bending)^[6]^ and 696 cm^-1^ (maleimide ring bending). (c) FTIR spectra of F-CSMG, exhibiting the same characteristic peaks as in F-HMG. (d) FTIR spectra of M-CSMG, showing the same maleimide-specific bands as in M-HMG. To enable meaningful quantitative comparison across samples, all spectra were normalized to the isopropyl group bending peak^[7]^ at 1386 cm⁻¹, which is inherent to the NIPAM backbone and remains unaffected by functionalization. The increasing intensities of these peaks with reagent concentration confirm successful and tunable incorporation of reactive groups via DMTMM-mediated coupling^[8]^.


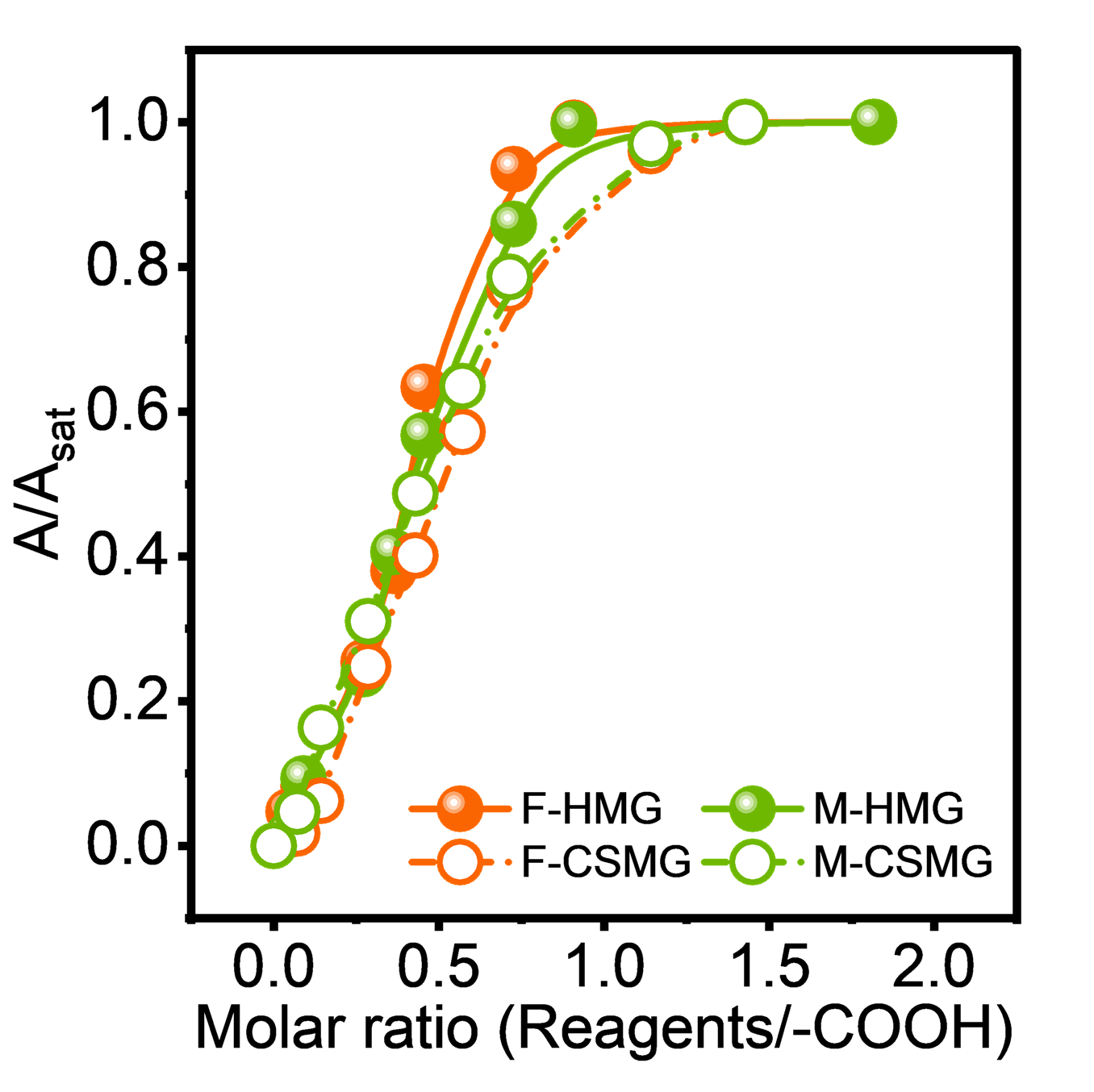


**Figure S6.** Conversion of carboxylic acid group by incorporation functional groups in microgels *via* FTIR peak integration. Normalized FTIR peak area (A/A_sat_) plotted against the reagent-to-carboxylic group molar ratio for F- (orange) and M- (green) MGs. Solid lines and symbols represent HMGs, while open symbols and dashed-dotted lines denote CSMGs. The C–H bending peak at 744 cm^-1^ was used for F-MGs, and the maleimide ring bending peak at 696 cm^-1^ was used for M-MGs. Peak areas were normalized to the saturated value (A_sat_) obtained at excess reagent concentration.

**Peak integration and normalization**:

The integrated area of the characteristic peak was calculated using the trapezoidal rule over:

- 700–776 cm^-1^ for the furan C–H bending peak
- 655–705 cm^-1^ for the maleimide ring bending peak

Each value was then normalized by the corresponding peak area at a 1.2:1 reagent-to-carboxylic acid molar ratio (A_sat_), which represents the saturated conversion level:

- Furan: A/A_sat_ = (area at 744 cm^-1^) / (area at 744 cm^-1^ at 1.2 ratio)
- Maleimide: A/A_sat_ = (area at 696 cm^-1^) / (area at 696 cm^-1^ at 1.2 ratio)

**Estimated coupling efficiency under reaction conditions used in the main text:**

Furan- and maleimide-functionalized microgels were prepared by DMTMM-mediated amidation in a 50:50 (v/v) ethanol: pH 5 MES buffer mixture. Reactions were conducted overnight (~16 h) at room temperature using DMTMM-to–COOH molar ratios of 0.27 for HMG and 0.36 for CSMG. After the reaction, samples were purified by five centrifugation-resuspension cycles in DI water.

Under these conditions, the estimated carboxyl group conversion determined from FTIR peak integration (Fig. S6) is:

• F-HMG: 0.254

• M-HMG: 0.237

• F-CSMG: 0.286

• M-CSMG: 0.310

These values represent the estimated fraction of carboxylic groups converted to Diels-Alder-reactive groups under the coupling conditions used throughout the study.

**
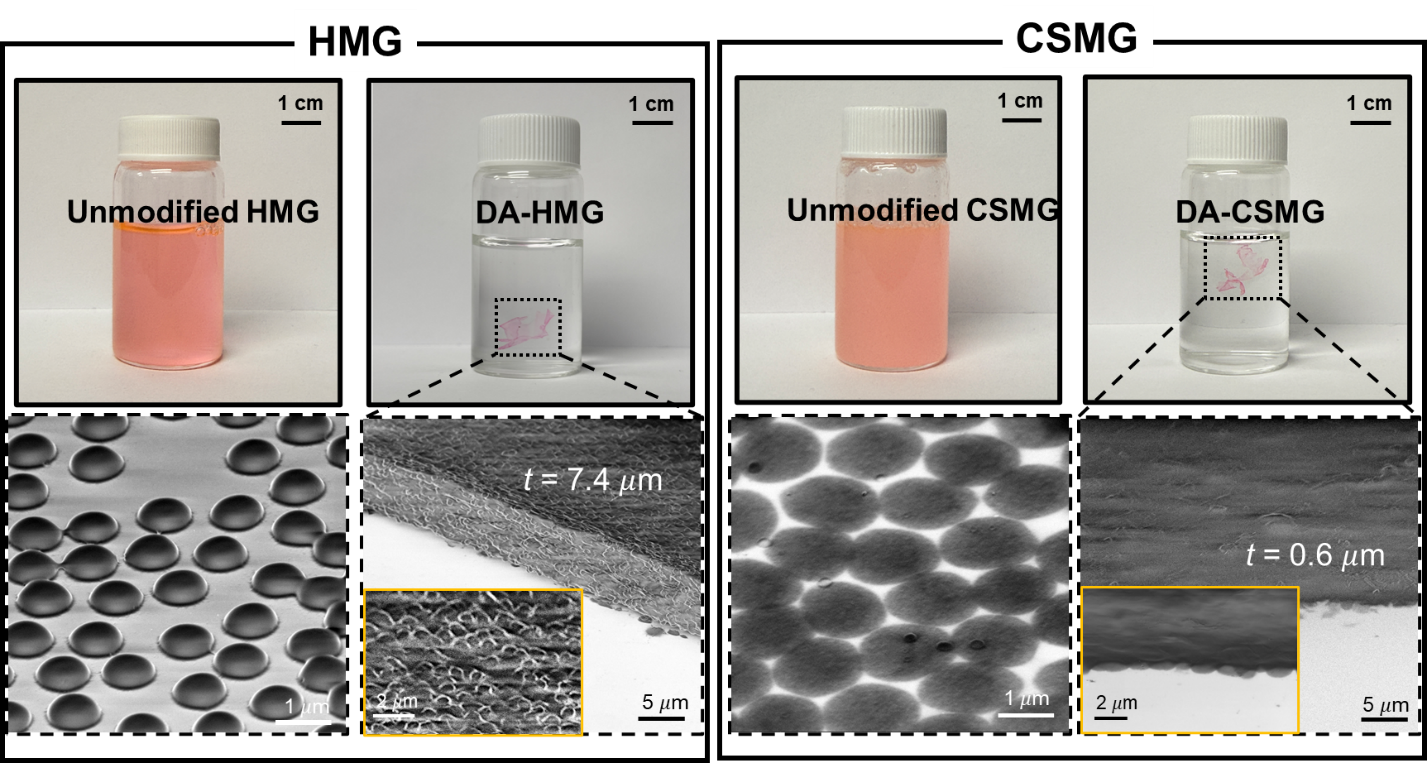
**

**Figure S7**. Formation and morphology of DA-MG films compared to unmodified MG. Photographs show that DA-MG films remain intact after immersion in water, while films made from unmodified MGs fully re-disperse. SEM images (ThermoFisher Helios G4, 1 keV, 21 pA, 50 ns dwell time, TLD, 50° incidence) are shown for both DA-MG films and unmodified MGs. Unmodified MGs appear as individually deposited particles on the silicon wafer substrate, while DA-MG films form a continuous, monolithic film with visible surface texture, suggesting interparticle connectivity and partial retention of original microgel morphology. While DA-CSMG films also remained intact in water, their dried SEM appearance was more featureless, lacking the distinct particle-like structure observed in DA-HMGs. This is likely due to the compliant nature of the PNIPAM-rich core in CSMGs, which causes greater deformation and flattering during drying and crosslinking, making the individual microgels less distinguishable in the final film.

**
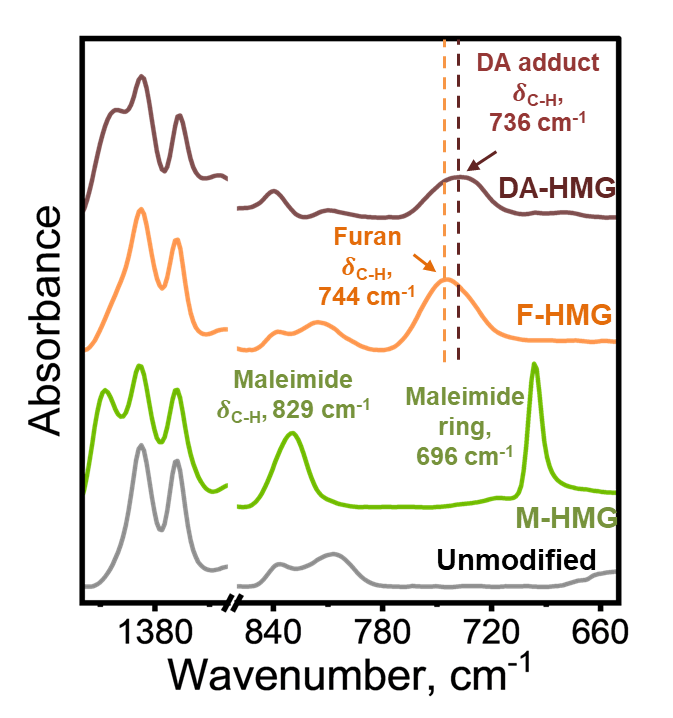
**

**Figure S8**. FTIR spectra confirming DA bond formation in DA-HMG films. ATR-FTIR spectra of dried DA-HMG films are compared with those of dried F-HMG, M-HMG, and unmodified HMG films. The disappearance of the maleimide ring bending peak at 696 cm^-1^ and a shift in the furan C–H bending peak (from 744 cm^-1^) in the spectrum of DA-HMG film indicate successful DA reaction between functionalized particles. All spectra were normalized to the isopropyl group bending band at 1386 cm^-1^ for quantitative comparison.

**
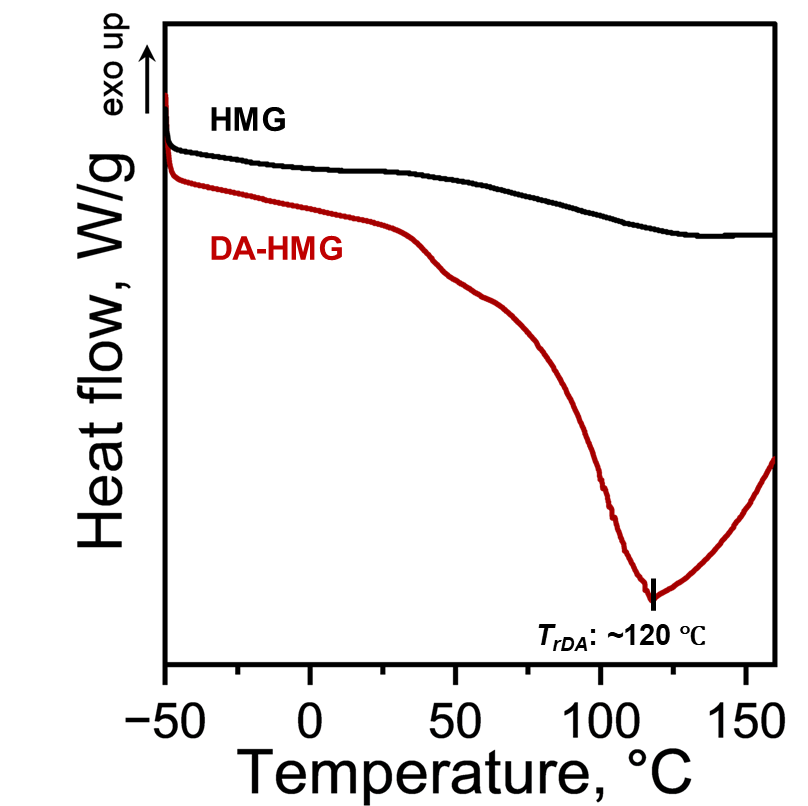
**

**Figure S9**. DSC thermogram of DA-HMG to determine DA bond dissociation temperature (T_rDA_). DSC was performed using a TA Instruments DSC 2500. Approximately 5-10 mg of dried DA-HMG film was weighed and sealed in Tzero aluminum pans with lids. The measurement was carried out under N_2_ atmosphere with a flow rate of 50 mL/min. The sample was heated from -50 ℃ to 160 ℃ at a ramp rate of 5 ℃/min. An endothermic peak centered around ~120 ℃ was observed, corresponding to the rDA bond dissociation of furan–maleimide adducts, confirming the presence of dynamic covalent bonds in the DA-HMG network.

**
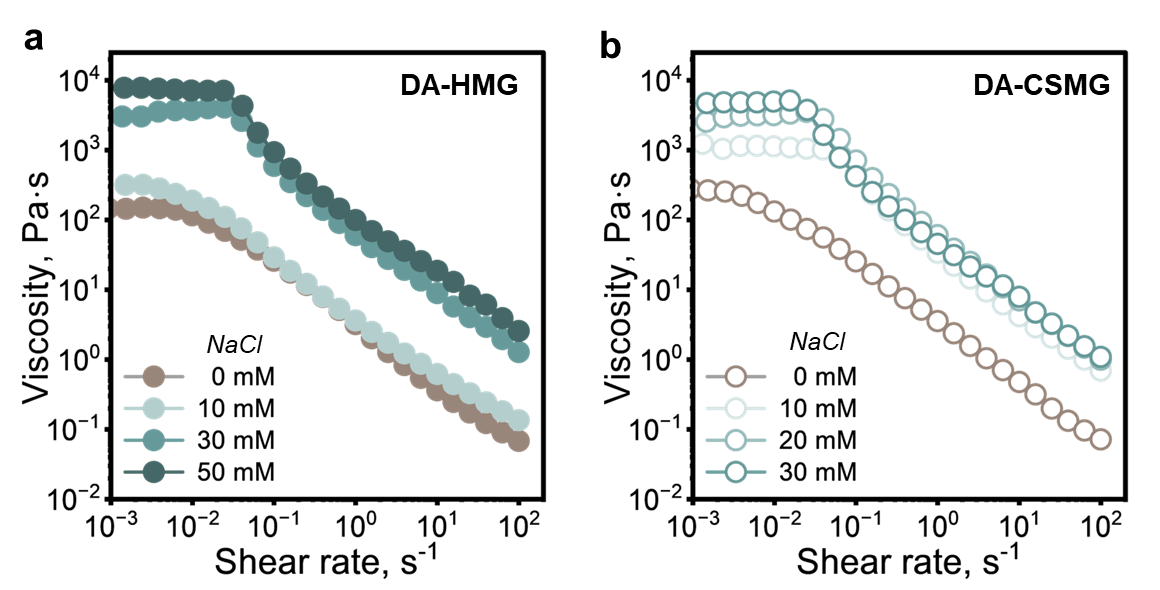
**

**Figure S10**. Viscosity of DA microgel inks with varying salt concentrations. (a) DA-HMG and (b) DA-CSMG dispersions exhibited characteristic shear-thinning behavior over a shear rate range of 0.001 to 100 s⁻¹. Each ink was prepared by centrifuging the respective microgel dispersions after addition of NaCl at varying concentrations (0–50 mM for DA-HMG and 0–30 mM for DA-CSMG dispersions). The increase in ionic strength reduced electrostatic repulsion between microgels, promoting physical jamming and enhancing viscosity.

**
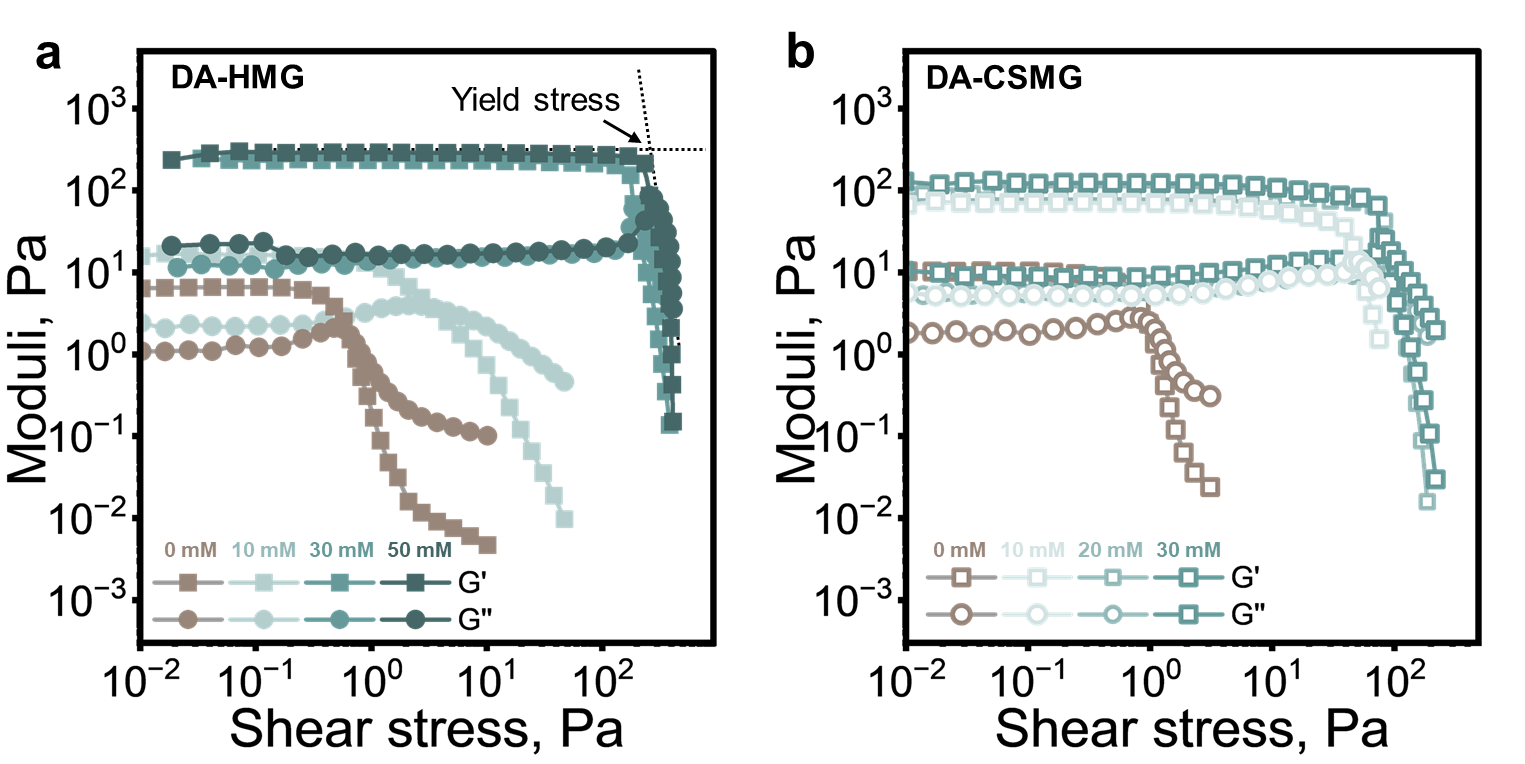
**

**Figure S11**. Oscillatory stress sweep tests to determine yield stress of DA microgel inks. Oscillatory stress sweep measurements (1 Hz, 0.01–1000 Pa) were performed on (a) DA-HMG and (b) DA-CSMG dispersions with varying NaCl concentrations to evaluate their yield stress. Storage modulus (G’, squares) and loss modulus (G’, circles) are plotted versus shear stress, with solid symbols representing DA-HMG and open symbols representing DA-CSMG dispersions. Yield stress was determined by identifying the stress at which G’ began to deviate from its initial plateau, indicating the onset of nonlinearity and yielding. The dashed lines on the G’ curves represent the linear regime used as reference for estimating the yield point.


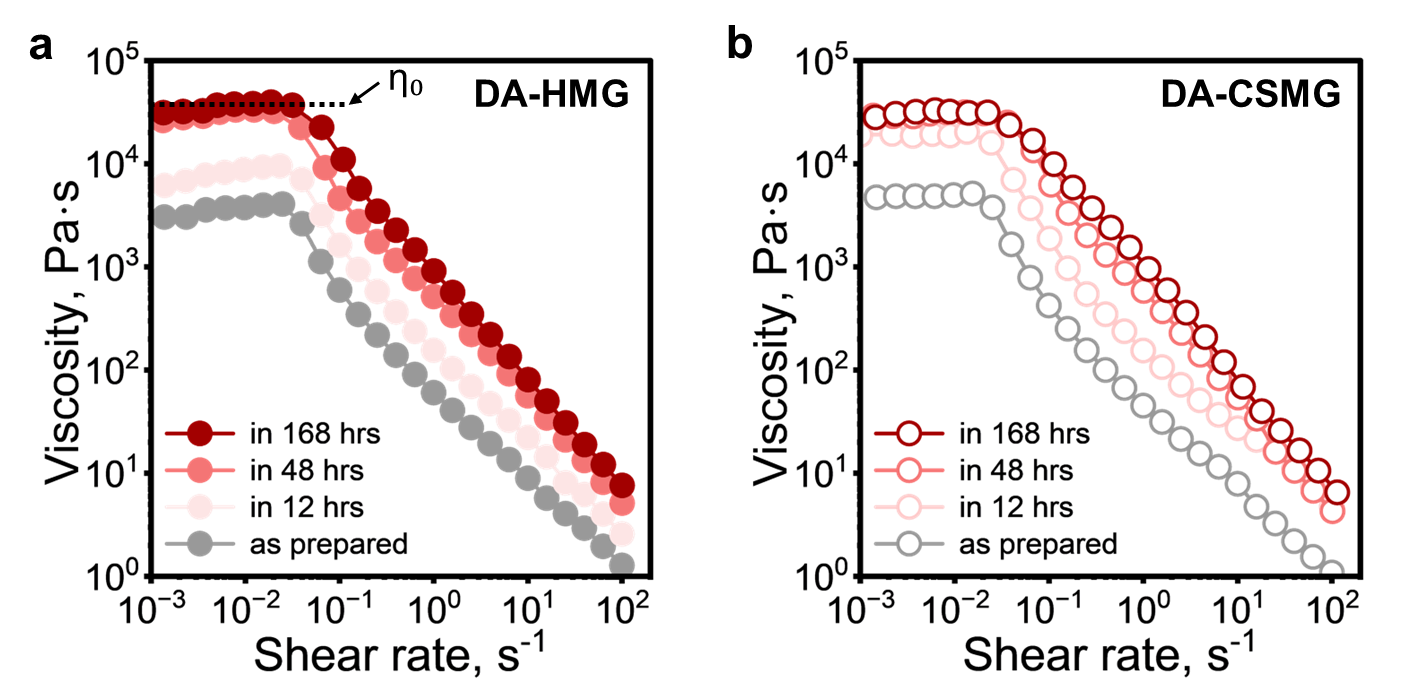


**Figure S12**. Viscosity profiles of (a) DA-HMG and (b) DA-CSMG inks measured over a shear rate range of 0.001–100 s^-1^ at different time points (0 to 168 h) after preparation with 30 mM NaCl and centrifugation. The zero-shear viscosity ($\eta_{0}$), defined as the viscosity in the low-shear regime (~0.01 s^-1^), was extracted by averaging values in this plateau region.


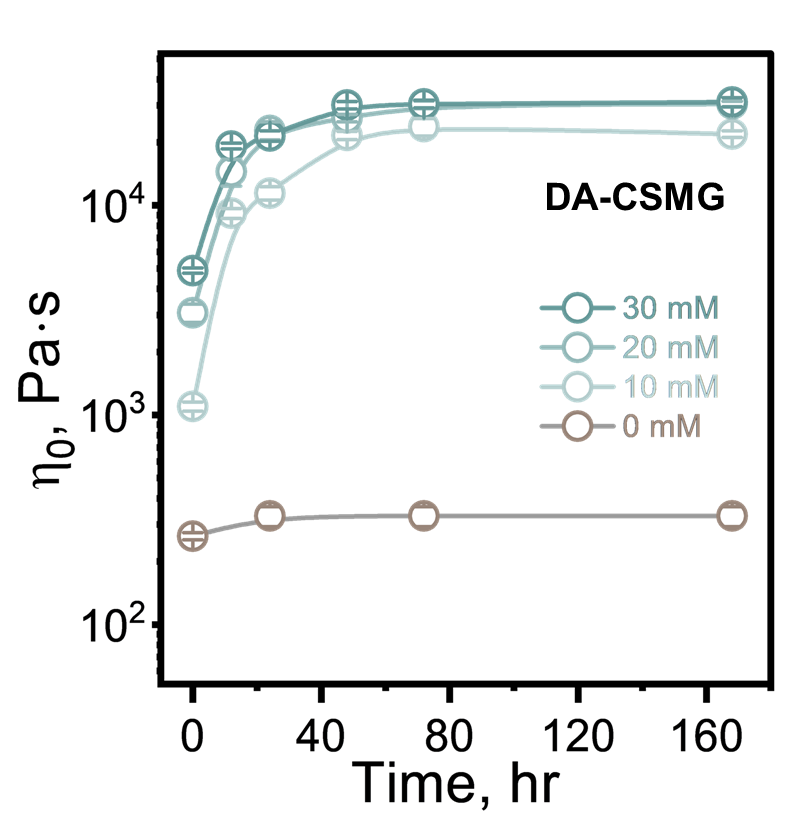


**Figure S13**. Time-dependent development of zero-shear viscosity, $\eta_{0}$, for DA-CSMG inks at varying NaCl concentrations. The inks were prepared by adding NaCl at concentrations of 0, 10, and 30 mM to the freshly modified DA-CSMG dispersions, followed by centrifugation to induce jamming. $\eta_{0}$ progressively increased with incubation time due to gradual formation of DA interparticle crosslinks, with faster kinetics observed at higher salt concentrations.


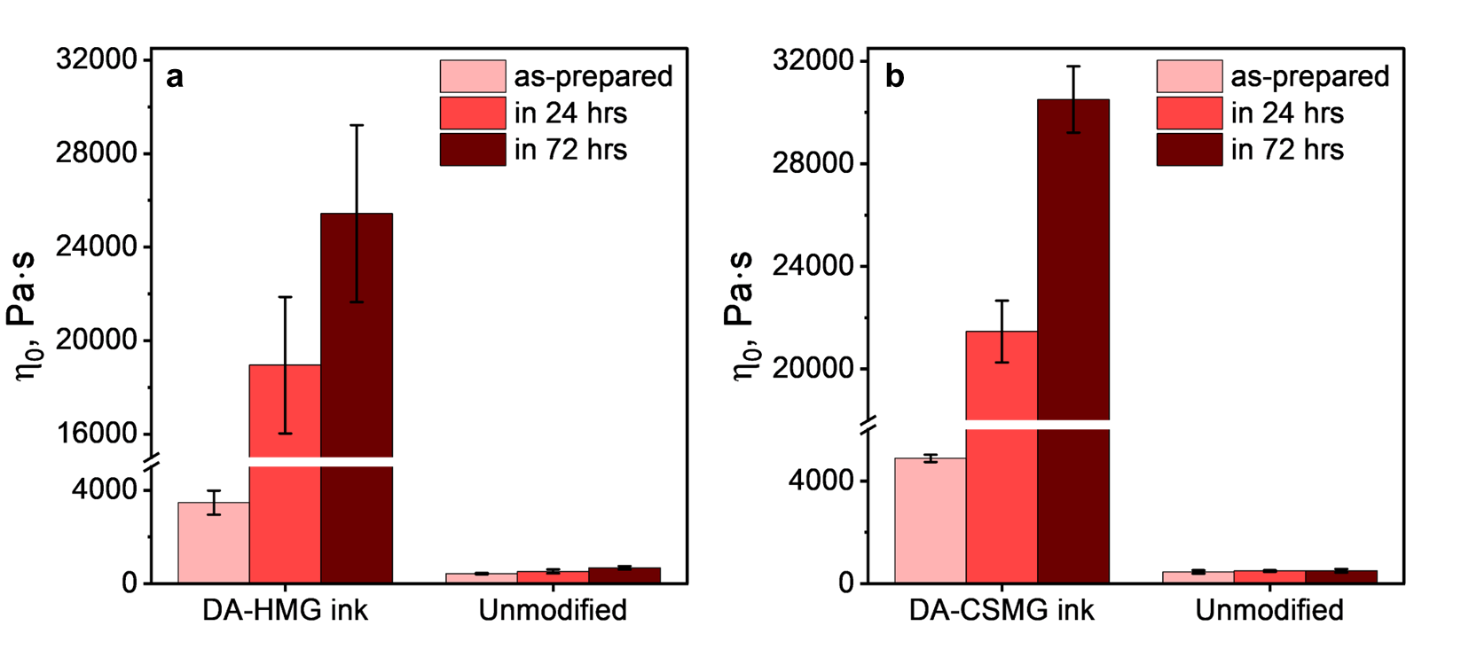


**Figure S14**. Time-dependent zero-shear viscosity (η₀) of DA-MG inks and unmodified MG dispersions at 30 mM NaCl, measured immediately after preparation (0 h), after 24 h, and after 72 h with (a) DA-HMG ink and HMG dispersions, and (b) DA-CSMG ink and CSMG dispersions.


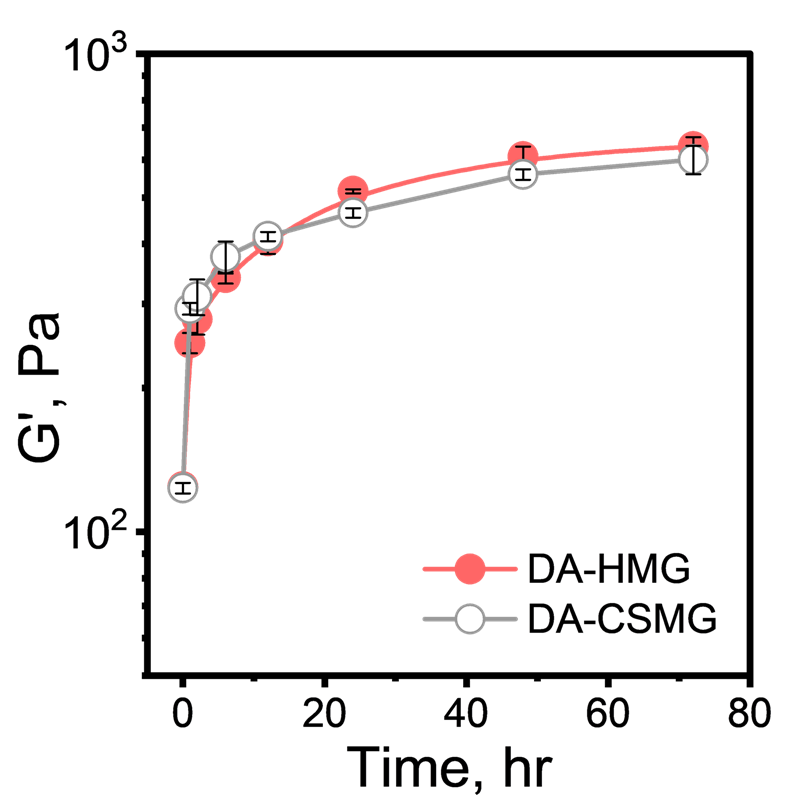


**Figure S15**. Time evolution of the average storage modulus (G’) for DA-HMG and DA-CSMG inks. G’ was measured as a function of incubation time (0-72 h) of DA-HMG and DA-CSMG inks prepared at 30 mM NaCl.


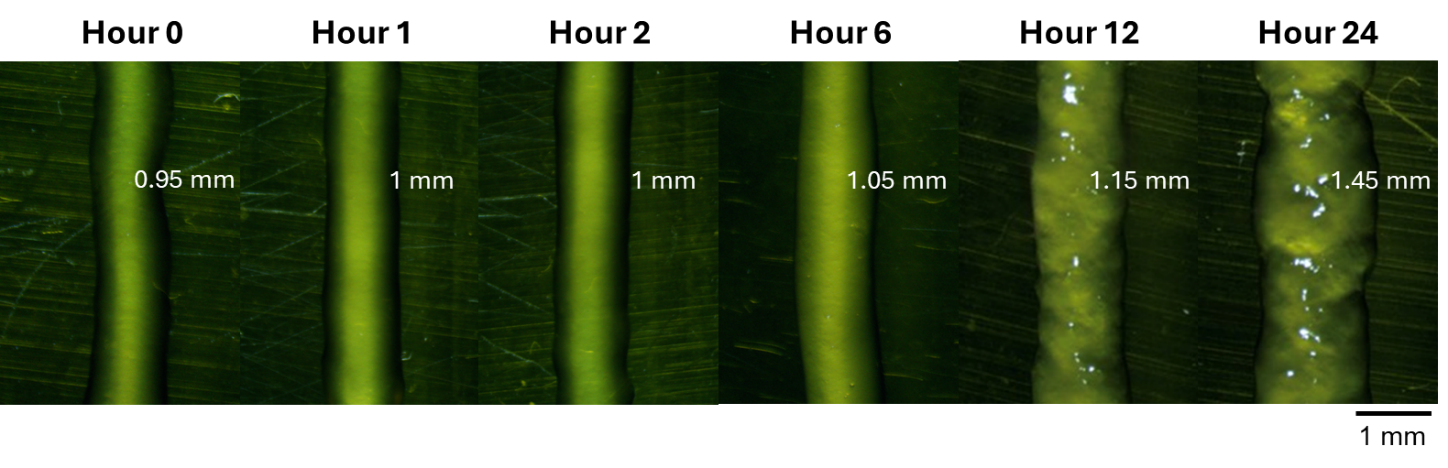


**Figure S16**. Extrudate filament stability and minimum printable feature size as a function of ink aging time. Extrudate filament widths for DA-MG inks aged for 0, 1, 2, 6, 12, and 24 h after preparation (30 mM NaCl, 22-gauge needle, 3 mm/s printing speed, 0.6 mm layer height). For each aging condition, the extrusion multiplier was tuned to produce a continuous extrudate while maintaining a fixed layer height. The minimum achievable filament widths were 0.95, 0.99, 0.99, 1.05, 1.15, and 1.45 mm for 0-24 h, respectively. Filament widths remained highly consistent over the first 6 h, indicating stable extrusion rheology within the practical pot-life window, while gradual DA-mediated network maturation at longer aging times (12–24 h) led to slightly thicker and grainier features due to increased microgel clustering. Scale bars: 1 mm.


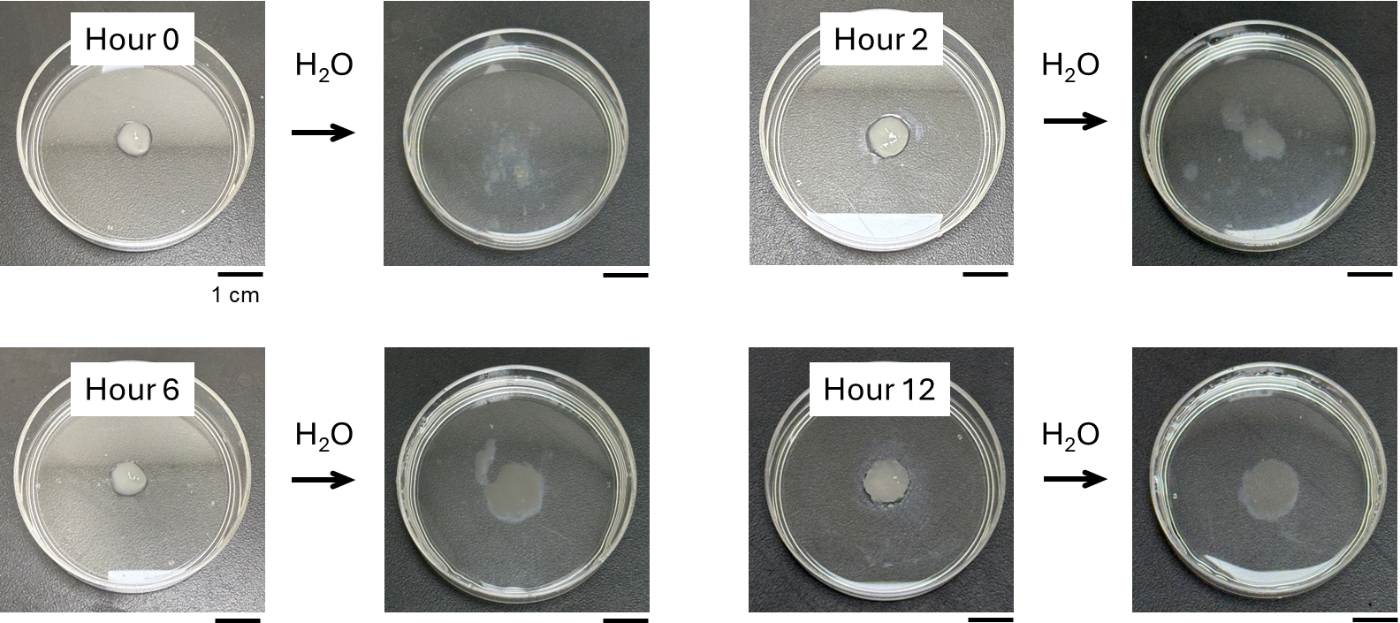


**Figure S17.** Stabilization of the syringe-extruded structures due to time-dependent network maturation. The simple structures deposited with DA-MG inks were incubated at ambient air conditions for 0, 2, 6, and 12 h prior to immersion in water. The immediately deposited constructs (0 h) disintegrated upon immersion, reflecting insufficient interparticle crosslinking. The structures which were aged for 2-6 h exhibited partial shape retention but displayed boundary erosion or fragmentation. By 12 h, the constructs completely maintained their shape. Scale bars: 1 cm.


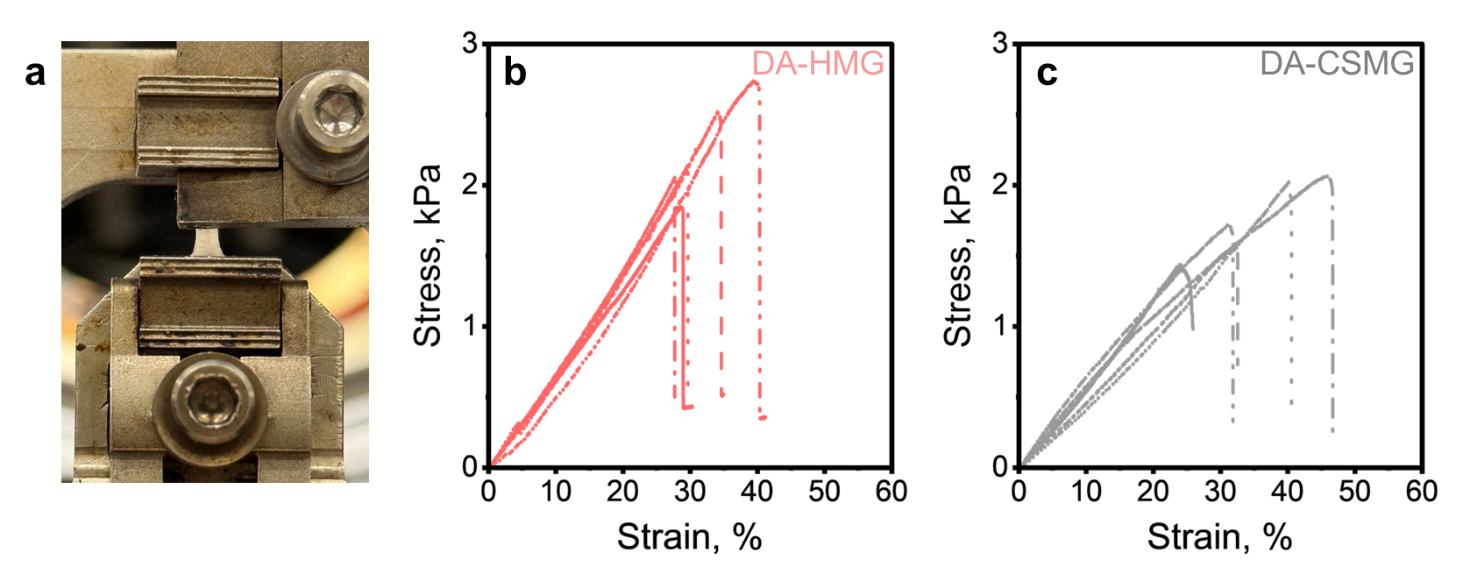


**Figure S18**. Tensile properties of DA-HMG and DA-CSMG assemblies. (a) Photographs of hydrogel strips (6 × 20 × 1 mm) during tensile testing. (b) Stress-strain curves (5 tests) for DA-HMG and (c) DA-CSMG networks using corresponding inks form at 30 mM NaCl.


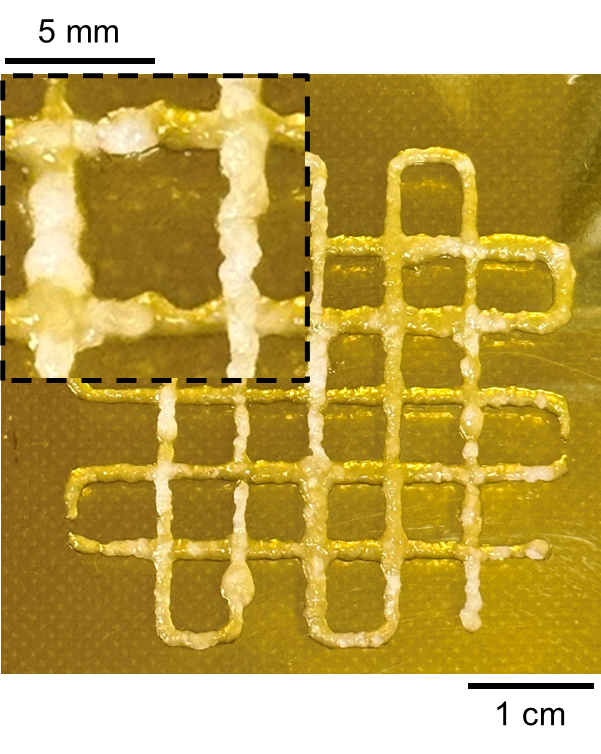


**Figure S19**. Printability of DA-HMG ink at 50 mM NaCl. The grid pattern was printed using DA-HMG ink formulated with 50 mM NaCl, under the same printing conditions as in Fig. 3e (22-gauge needle, 3 mm/s printing speed, 0.6 mm layer height). The printed filaments exhibited surface roughness, local thickening, and poorly defined pore due to pre-extrusion aggregation and excessive cohesion at this salt concentration. The scale bar is 1 cm (global), 5 mm (enlarged).


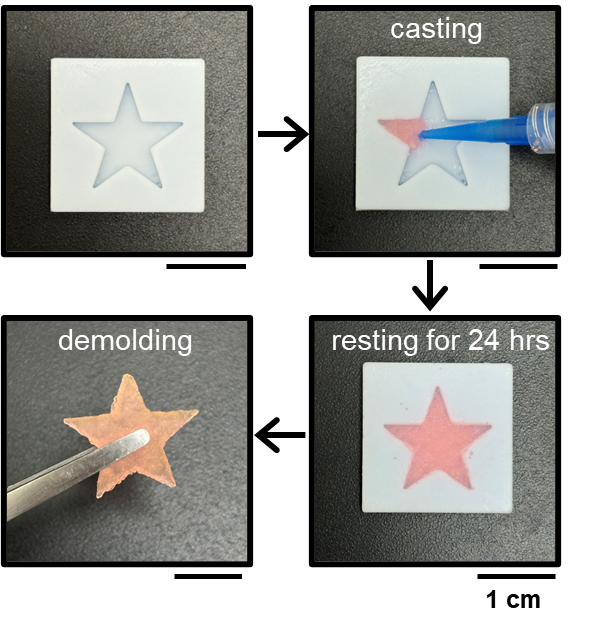


**Figure S20**. Moldability and cohesive integrity of DA-HMG inks upon casting. DA-HMG ink (dyed with Rhodamine 6G for visual contrast) was injected into a star-shaped mold (1 mm-thick), incubated at 4 °C for 24 h, and then demolded. The resulting hydrogel retained its molded shape and was mechanically robust enough to be handled with tweezers, demonstrating cohesive integrity suitable for free-standing applications.

**
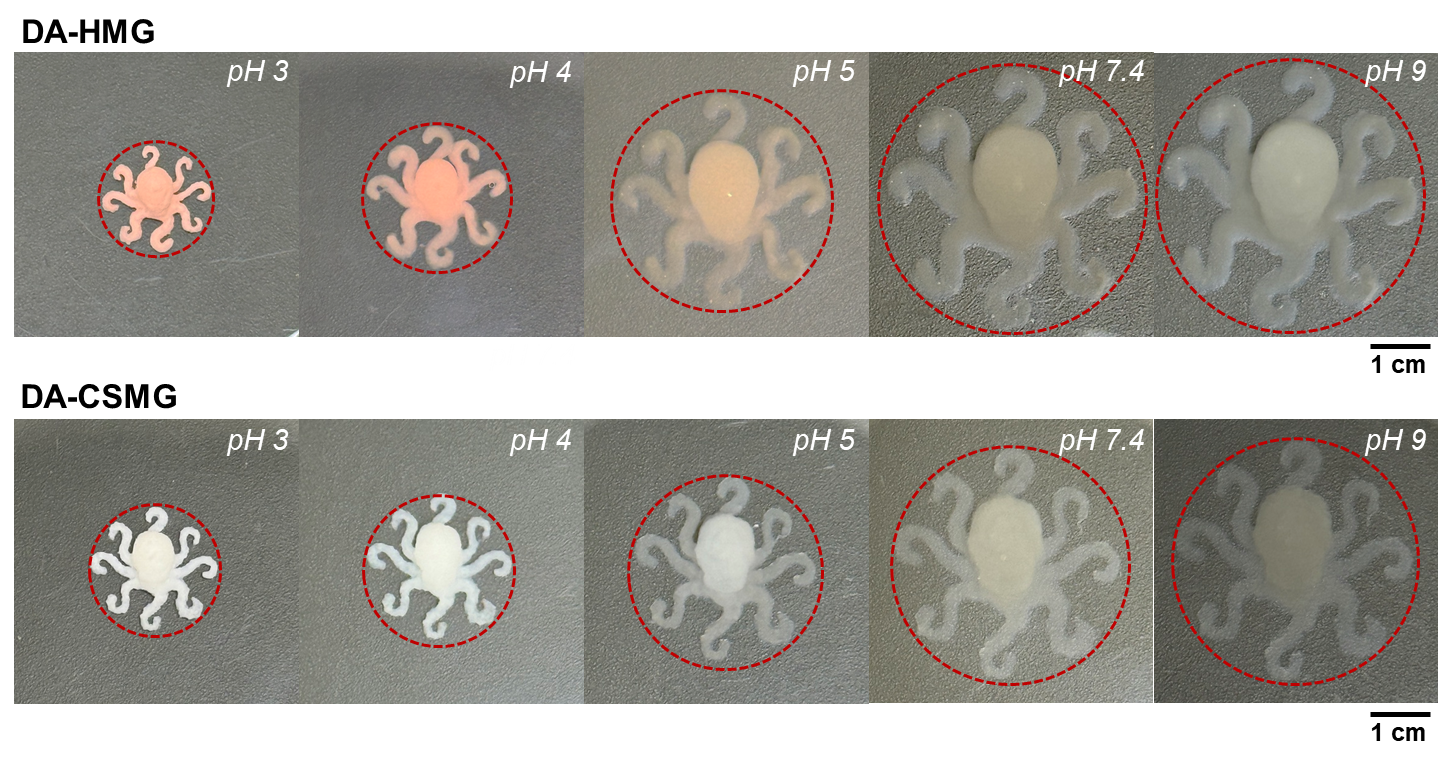
**

**Figure S21**. pH-responsive swelling of 3D printed DA-MG octopus assemblies in aqueous environments. Sequential images of 3D printed octopus structures made from DA-HMG (top panel) and DA-CSMG (bottom panel) during a pH sweep from 3 to 9. Both assemblies preserved their overall architecture while undergoing swelling–deswelling behavior in response to pH changes, highlighting the robustness and stimuli-responsive nature of the interlinked microgel networks.

**
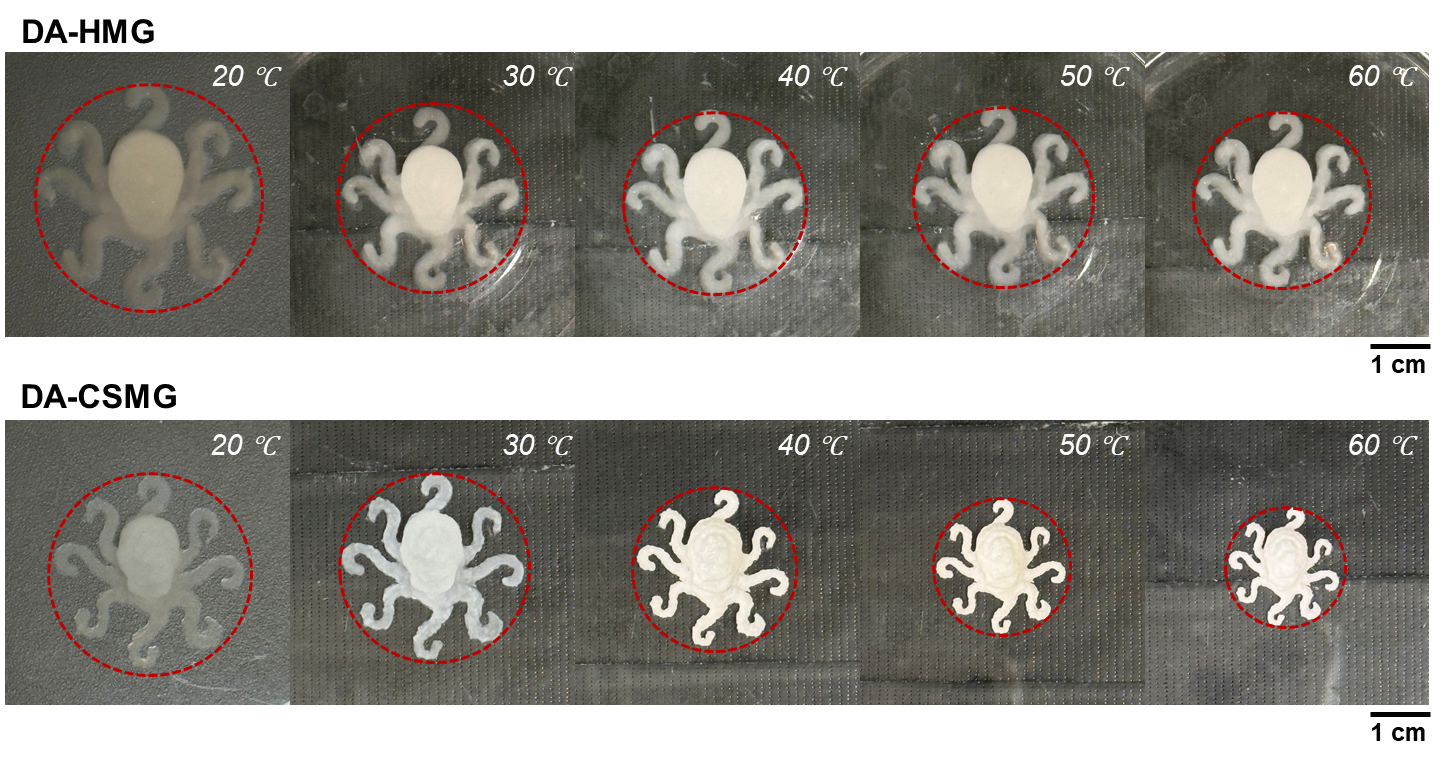
**

**Figure S22**. Temperature-responsive behavior of 3D printed DA-MG assemblies. 3D printed octopus-shaped constructs made from DA-HMG (top panel) and DA-CSMG (bottom panel) were subjected to temperature changes from 20 ℃ to 60 ℃ in pH 5 buffer. Images show the deswelling behavior driven by thermoresponsive PNIPAM segments. DA-CSMG exhibited pronounced shrinkage due to the PNIPAM core, while DA-HMG showed minimal size change but increased turbidity, indicating internal phase transition. All constructs retained their overall shape and structural integrity throughout thermal response, highlighting the robustness of the DA-interlinked microgel framework.


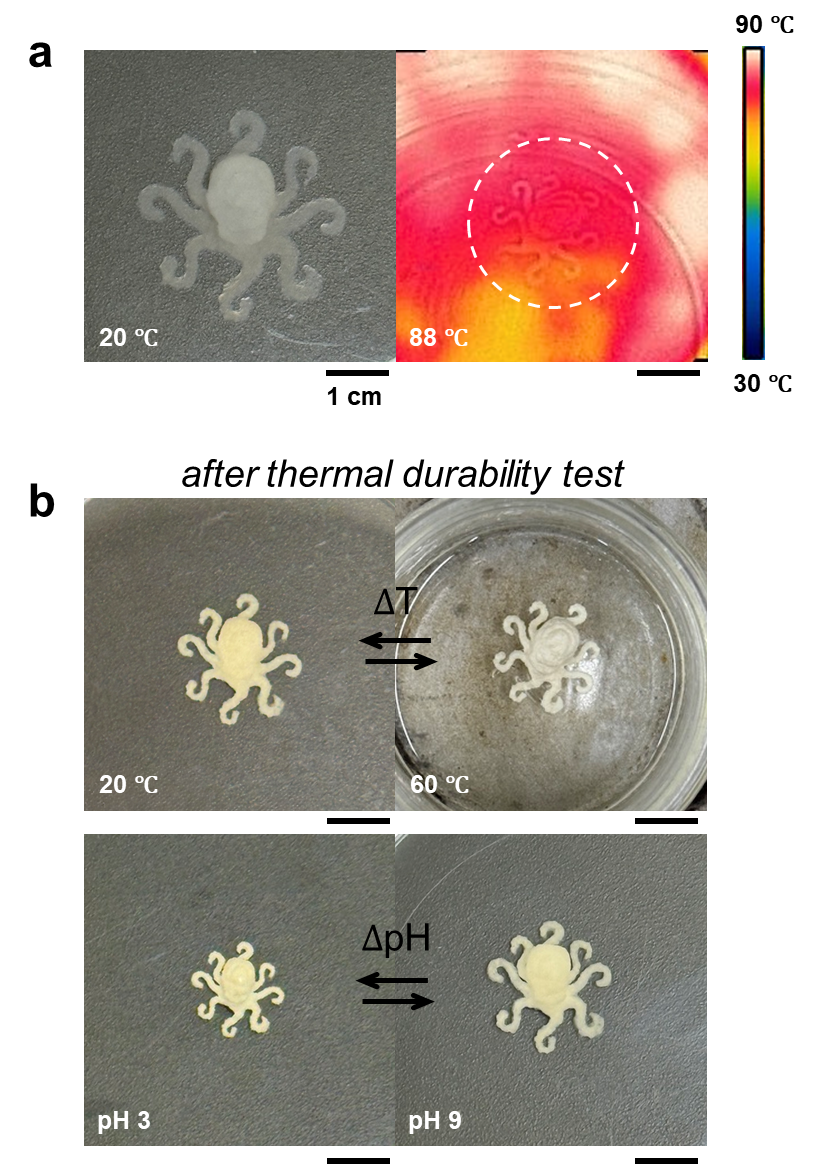


**Figure S23.** Thermal durability and network reconfiguration of DA-CSMG printed constructs after extended exposure to 88 °C. (a) DA-CSMG octopus structure before and after immersion in water at 88 °C for 24 h. The construct remained intact without fragmentation, confirming cohesive network stability below the rDA peak temperature ($\approx$120 °C). Prolonged heating induced $\approx$63% permanent reduction of its size due to thermally driven bond exchange and reconfiguration of the dynamic network. (b) Temperature and pH-induced actuation of the same construct after thermal conditioning. Irreversible network reconfiguration and densification resulted in reduced response amplitudes relative to the fresh sample. After conditioning, the construct exhibits $\approx$10% reversible contraction between 20 °C and 60 °C (compared to $\approx$50% for a fresh sample) and $\approx$40% reversible swelling between pH 3 and 9 (compared to $\approx$90% for a fresh sample). Scale bars: 1 cm.

**
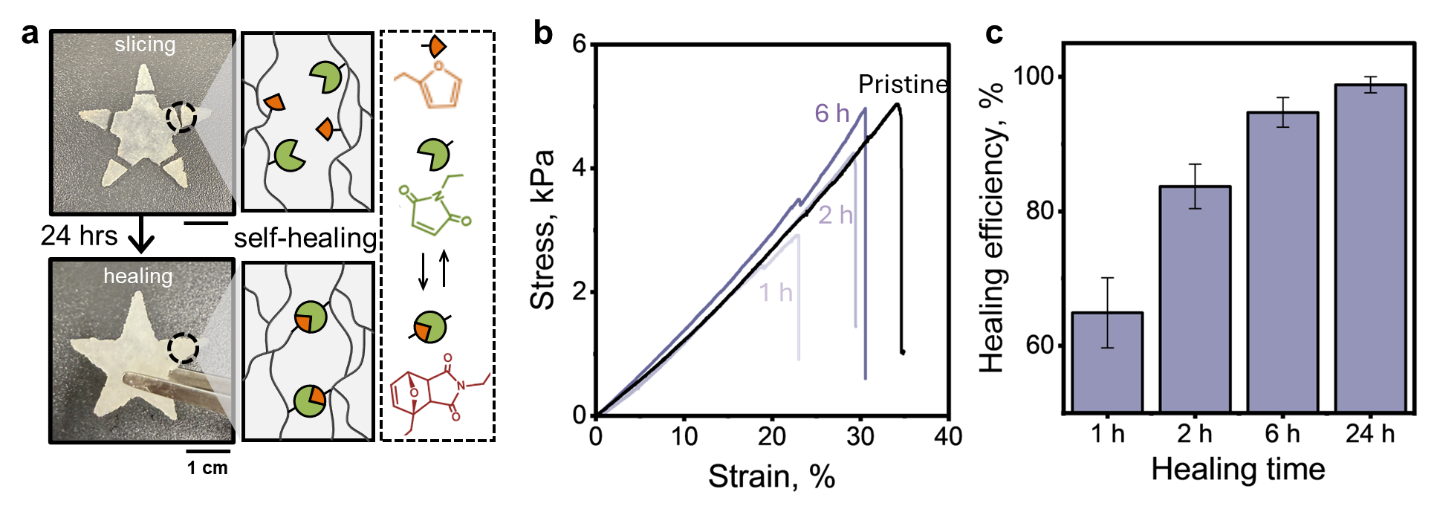
**

**Figure S24**. Self-healing behavior of DA-HMG *via* DA covalent adaptable bonds. (a) A star-shaped DA-HMG construct was cut into fragments and the pieces were manually brought into contact and incubated at 4 °C for 24 h. The healed structure visibly rejoined without the need for external stimuli, demonstrating self-healing enabled by the reformation of DA bonds. Schematics illustrate the dissociated (pre-healing) and re-associated (post-healing) states of interparticle dynamic covalent bonding. (b) Representative stress-strain curves for the pristine and self-healed DA-HMG strips after 1, 2, and 6 h of healing, showing progressive mechanical recovery. (c) Healing efficiency quantified via the ratio of the ultimate tensile strength of the healed samples to the pristine value. Error bars represent standard deviations (n = 5).

**
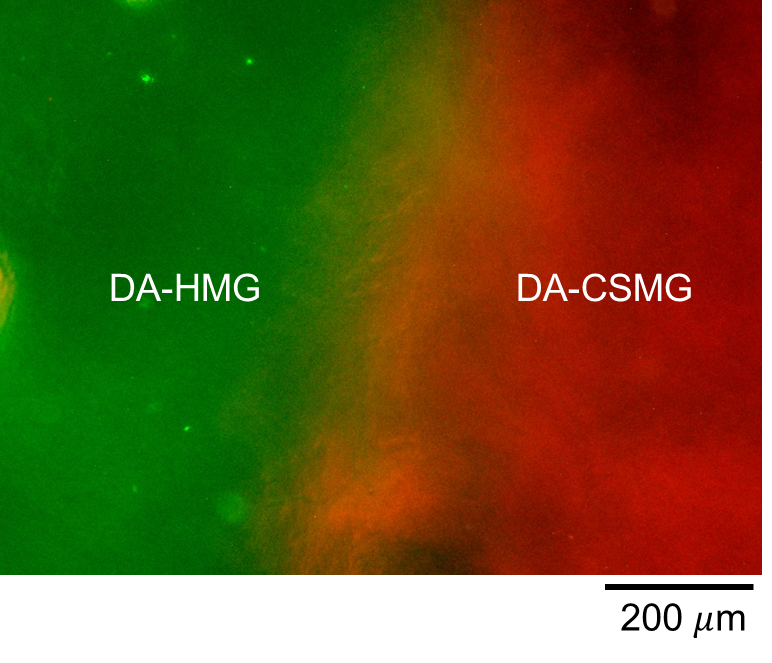
**

**Figure S25**. Dual-color confocal fluorescence image demonstrating the integrity of the interface between DA-HMG and DA-CSMG domains after DA-mediated network maturation. HMG microgels were labeled with AZDye 488 Cadaverine (green) and CSMG microgels with AZDye 594 Cadaverine (red) via DMTMM-mediated amidation (0.1 mol%). Each labeled F-MG dispersion was mixed with an equivalent amount of unlabeled M-MG, processed into clickable inks using salt-assisted jamming, and deposited in a two-layer multimaterial geometry. After 24 h of ambient maturation, cross-sectional fluorescence images were acquired at 5$\times$ magnification, with each channel collected separately using the emission filters centered at 520 nm for AZDye 488 and 610 nm for AZDye 594. The two channels were subsequently overlaid using ImageJ. Scale bar: 200 $\mu$m.

**Thermal Durability and High-Temperature Network Reconfiguration**

To assess long-term thermal durability and address the potential for creep or structural drift near the retro-Diels–Alder (rDA) transition, a DA-CSMG octopus construct was immersed in water at 88 °C for 24 h, a significantly more demanding condition than typical hydrogel operating environments. This temperature lies above the LCST of PNIPAM and near the onset of the rDA endotherm ($\approx$80 °C), yet remains below the dissociation peak ($\approx$120 °C). Remarkably, the printed construct remained intact after 24 h at 90 °C without fragmentation, delamination, or loss of overall shape (Fig. S23a). This confirms that the DA-crosslinked interparticle network preserves cohesive integrity under extended exposure to elevated temperatures. Such stability would align with prior observations that, between $\approx$60–120 °C, Diels-Alder networks undergo *endo*-to-*exo* isomerization and bond reshuffling rather than rapid, complete bond dissociation.^[9]^ Following the 24 h dwell, the construct displayed permanent shrinkage of $\approx$63% in linear dimension, indicating thermally driven network reconfiguration. We attribute this compaction to bond-exchange-enabled plasticization, allowing the network to adopt a denser topology under prolonged thermal stress. Importantly, even after this significant topological rearrangement, the structure remained cohesive and responsive while the actuation amplitude was reduced. Specifically, the thermally conditioned construct showed $\approx$10% reversible contraction between 20 °C and 60 °C (compared to ~50% for the fresh sample) and $\approx$40% reversible swelling between pH 3 and 9 (compared to $\approx$90% for the fresh sample; Fig. S23b). The reduced amplitude reflects the densification of post-annealed network rather than degradation or loss of interparticle connectivity.

These results demonstrate that DA-crosslinked microgel assemblies maintain structural integrity in aqueous environments up to at least $\approx$90 °C for 24 h, while undergoing permanent network reconfiguration leading to attenuates actuation amplitude. This high-temperature conditioning regime can be leveraged for tuning the dimensions of the printed responsive constructs, while for applications requiring large-amplitude actuation should be limited to temperatures not exceeding 60 ℃.

**References:**

[1] Narupai, B., Smith, P. T., Nelson, A., “4D printing of multi‐stimuli responsive protein‐based hydrogels for autonomous shape transformations”, *Advanced Functional Materials*, 31 (2021): 2011012

[2] Arsuffi, B., Siqueira, G., Nyström, G., Titotto, S., Magrini, T., Daraio, C., “Programmable multi‐responsive nanocellulose‐based hydrogels with embodied logic”, *Advanced Functional Materials*, 34 (2024): 2409864

[3] Downs, F. G., Lunn, D. J., Booth, M. J., Sauer, J. B., Ramsay, W. J., Klemperer, R. G., Hawker, C. J., Bayley, H., “Multi-responsive hydrogel structures from patterned droplet networks”, *Nature Chemistry*, 12 (2020): 363

[4] Liu, J., Huang, Y.-S., Liu, Y., Zhang, D., Koynov, K., Butt, H.-J., Wu, S., “Reconfiguring hydrogel assemblies using a photocontrolled metallopolymer adhesive for multiple customized functions”, *Nature Chemistry*, 16 (2024): 1024

[5] Mishra, P., Hull, A. W., Barnum, T. J., McGuire, B. A., Field, R. W., “Chirped-pulse Fourier-transform millimeter-wave rotational spectroscopy of furan in its v10 and v13 excited vibrational states”, *Journal of Molecular Spectroscopy*, 388 (2022): 111686

[6] Aguiar, E. C., Da Silva, J. B. P., Ramos, M. N., “A theoretical study of the vibrational spectrum of maleimide”, *Journal of Molecular Structure*, 993 (2011): 431

[7] Shah, L. A., Farooqi, Z. H., Naeem, H., Shah, S. M., Siddiq, M., “Synthesis and characterization of poly (N-isopropylacrylamide) hybrid microgels with different cross-linker contents”, *Journal of The Chemical Society of Pakistan*, 35 (2013): 1522

[8] Kunishima, M., Kawachi, C., Hioki, K., Terao, K., Tani, S., “Formation of carboxamides by direct condensation of carboxylic acids and amines in alcohols using a new alcohol-and water-soluble condensing agent: DMT-MM”, *Tetrahedron*, 57 (2001): 1551

[9] Gandini, A., “The furan/maleimide Diels–Alder reaction: A versatile click–unclick tool in macromolecular synthesis”, *Progress in Polymer Science*, 38 (2013): 1
